# Molecular changes induced by the curcumin analogue D6 in human melanoma cells

**DOI:** 10.1186/1476-4598-12-37

**Published:** 2013-05-04

**Authors:** Carla Rozzo, Manuela Fanciulli, Cristina Fraumene, Antonio Corrias, Tiziana Cubeddu, Ilaria Sassu, Sara Cossu, Valentina Nieddu, Grazia Galleri, Emanuela Azara, Maria Antonietta Dettori, Davide Fabbri, Giuseppe Palmieri, Marina Pisano

**Affiliations:** 1 Biomolecular Chemistry Institute, National Research Council of Italy. Traversa La Crucca, 3. 07100, Sassari, ITALY; 2 Porto Conte Ricerche srl. Località Tramariglio, Alghero, (SS), ITALY

## Abstract

**Background:**

In a previous report, we described the *in vitro* and *in vivo* antiproliferative and proapoptotic activity of a hydroxylated biphenyl (D6), a structural analogue of curcumin, on malignant melanoma and neuroblastoma tumours. In this paper, we investigated the molecular changes induced by such a compound, underlying cell growth arrest and apoptosis in melanoma cells.

**Results:**

To shed light on the mechanisms of action of D6, we firstly demonstrated its quick cellular uptake and subsequent block of cell cycle in G2/M phase transition. A gene expression profile analysis of D6-treated melanoma cells and fibroblasts was then carried out on high density microarrays, to assess gene expression changes induced by this compound. The expression profile study evidenced both an induction of stress response pathways and a modulation of cell growth regulation mechanisms. In particular, our data suggest that the antiproliferative and proapoptotic activities of D6 in melanoma could be partially driven by up-regulation of the p53 signalling pathways as well as by down-regulation of the PI3K/Akt and NF-kB pathways. Modulation of gene expression due to D6 treatment was verified by western blot analysis for single proteins of interest, confirming the results from the gene expression profile analysis.

**Conclusions:**

Our findings contribute to the understanding of the mechanisms of action of D6, through a comprehensive description of the molecular changes induced by this compound at the gene expression level, in agreement with the previously reported anti-tumour effects on melanoma cells.

## Background

Melanoma is the most aggressive form of skin cancer. Its incidence and mortality have risen dramatically in all developed countries during the last half century [1]. Although most cases of melanoma are diagnosed early and surgically resected, later stages of this tumour have very poor survival rates because of the lack of available effective therapies [1-3]. Recently, promising therapeutic approaches for melanoma management have been introduced into the clinical practice, based mostly on the use of small-molecule inhibitors directed against oncogenic molecular targets as well as on immunotherapy [4-7]. However, a high molecular heterogeneity of melanoma tumours and a complex network of proliferation and survival pathways involved in its pathogenesis have been reported [8,9]. For this reason, there is a growing interest in seeking pharmacological agents that could target multiple gene products in order to interfere, at different levels, with pathogenetic pathways in melanoma. During the last decades, several dietary agents have been reported to exert anticancer activity. They commonly show multifaceted effects on cancer cells by inducing molecular changes related to different mechanisms of carcinogenesis: proliferation, apoptosis, invasion, and metastasis [10]. An innovative therapeutic approach to manage melanoma may be represented by the introduction into clinical trials of naturally occurring compounds (such as eugenol, resveratrol, green tea, curcumin and other), whose antiproliferative and/or proapoptotic activity against malignant melanoma - in both *in vitro* and *in vivo* models - has been already demonstrated [11]. Among them, curcumin, a polyphenol extracted from the rhizome of the plant *Curcuma longa*, has been frequently reported to exert promising anticancer activity on several tumours [12]. This molecule is highly pleiotropic, is able to enter cells [13], and interacts with numerous targets [14]. Strong evidence demonstrated that curcumin inhibits proliferation, invasion, angiogenesis, and metastasis in several types of cancer through interaction with multiple cell signalling proteins (reviewed in [15,16]). Recently, curcumin has been shown to exert a good antiproliferative activity by inducing apoptosis in malignant melanoma [17]. One of the most important pathway involved in the curcumin antitumour activity is the nuclear factor-kB (NF-kB) pathway [18], particularly in melanoma cells. Indeed, curcumin is able to suppress the activation and phosphorylation of the inhibitor of NF-kB alpha (IkBa) by inhibiting the IkB kinase (IKK) and NF-kB activity in human melanoma cell lines [19,20]. Moreover, curcumin induces cell apoptosis and cell cycle arrest in G2/M phase in melanoma, through up-regulation of p53, p21, p27 and checkpoint kinase 2 [19].

Recently, our group has synthesized a new curcumin-related biphenyl structure (an αβ unsaturated ketone called D6) whose antiproliferative and proapoptotic activities on melanoma cell lines were more effective, rapid and selective than those induced by curcumin. The D6 compound was proved to promote apoptosis in melanoma cells through the mitochondrial intrinsic pathway [21]. *In vivo* assays on mouse models confirmed the potential of D6 against melanoma, showing a significant reduction of the tumour mass growth as compared to untreated control [21].

To investigate the mechanisms of action of the D6-curcumin analogue against melanoma at the molecular level, we here studied its cellular uptake and its influence on cell cycle progression. Finally, a gene expression profile analysis of D6-treated melanoma cell lines was carried out on high density microarrays, in order to explore the molecular pathways activated after D6 enters cells. This genomic technology is useful to dissect the molecular changes occurring inside cancer cells, and it is well documented for malignant melanoma [22,23]. In our study, the LB24Dagi (LB24) primary melanoma cell line was selected for all the analyses, because it had been previously demonstrated to be the most sensitive line to D6 treatment among tested ones [21]. Several molecular changes that can justify the antiproliferative and proapoptotic properties of D6 on melanoma cells and likely contribute to its anti-tumour effect have been here presented and discussed.

## Results

### D6 enters melanoma cells

**Figure 1:**
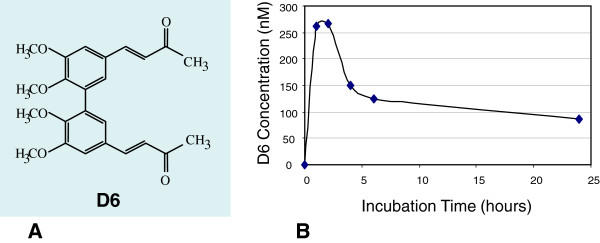
**D6 cellular uptake. A**: molecular structure of the curcumin-related biphenyl compound, the αβ-unsaturated ketone D6 (*3E*,*3*'*E*)-*4*,*4*'-(*5*,*5*',*6*,*6*'-*tetramethoxy*-[*1*,*1*'-*biphenyl*]-*3*,*3*'-*diyl*)*bis*(*but*-*3*-*en*-*2*-*one*); **B**: intracellular D6 mean concentrations following incubation of LB24 cells with 10 μM D6 for the indicated times. Mean values were calculated by the LC/MS method.

To verify the ability of D6 (Figure 1A) to enter melanoma cells, as demonstrated for curcumin in different cancer cells [13], we performed cellular uptake studies. After a 24 hours time course treatment, D6 cellular uptake was estimated by LC-MS on methanol cell lysates, as described in Methods. Comparison of D6 peak area for each sample to a calibration curve allowed us to calculate intracellular D6 concentration at different times. Data reported in Figure 1B show that the highest cellular D6 concentration (about 270 nM, corresponding to approximately 600 pmoles/10^6^ cells) was reached two hours after treatment. These results indicated that D6 presents the same time of uptake of curcumin in other cancer cells and is able to enter melanoma cells about 15 folds more efficiently than curcumin itself (34–44 pmoles/10^6^ cells [13]).

### D6 blocks cell cycle at G2/M transition

**Figure 2:**
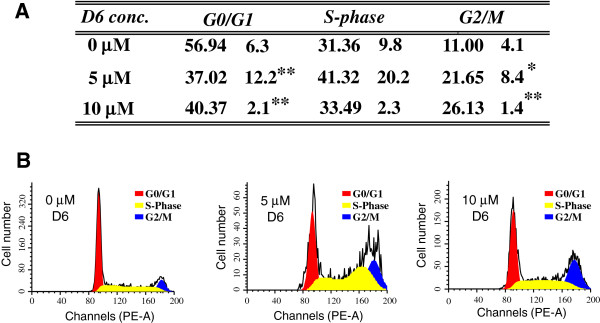
**Cell cycle progression analysis.** Cytofluorimetric analysis results from LB24 cells, untreated or treated with D6 for 24 hours. **A:** the table summarizes the percentage mean values ± SD of each cell cycle phase cell populations, obtained in three independent experiments. T-test was performed independently for each phase considering untreated (0 μM) versus treated (5 and 10 μM D6 respectively) cells. * P< 0.05 , ** P< 0.001; **B:** DNA content frequency histograms representing cells from untreated (0 μM) or 5 and 10 μM D6 treated LB24 cells. The histograms are referred to one, representative of three independent experiments.

To evaluate the effect of D6 treatment on melanoma cell cycle progression, we performed flow cytofluorimetric analysis on LB24 cells treated with either 5 or 10 μM D6 for 24 hours and stained with propidium iodide, as described in Methods. Results obtained are summarized in Figure 2. A significant enrichment in G2/M cell populations was observed at both 5 μM (21.65%) and 10 μM (26.13%) concentrations of D6 treatment, as compared to untreated cells (11.0%) (P < 0.05 and < 0.001 respectively) (Figure 2A). As a consequence, a significant reduction of G0/G1 phase cell population confirms the cell cycle arrest in G2 as an effect of melanoma cells exposure to D6. Figure 2B shows representative cell cycle histograms with a consistent increase in S phase cell number, indicating an accumulation of cells that do not trespass the G2/M checkpoint. Altogether, such findings strongly suggest that block of cell cycle progression may represent one of the mechanisms by which D6 inhibits melanoma cells growth (as previously observed by our group [21]).

### D6 treatment induces transcriptional changes in melanoma cells and normal fibroblasts

To analyze gene expression modifications induced by D6 treatment on melanoma cells, we carried out gene expression profile analyses on LB24 primary melanoma cell line, either treated or not (negative control) with 10 μM D6, using high density microarrays (see Methods). Same analysis was performed on human fibroblasts cells (BJ cell line) as normal control, which have been previously demonstrated to be insensitive to D6 [21].

Gene expression results were firstly filtered, in order to avoid analysis of background detection values (see Methods). Overall, 18,798 probes, representing the effective gene expression profiles of cell populations examined, were selected to perform the statistical analysis. This allowed the identification of gene transcripts whose expression was modulated by D6 treatment in each of the two cell types. Gene expression values obtained from D6 treated cells were compared with those obtained from untreated cells and fold change values (FC) were determined. For each cell population, probes showing FC values above 2 (double intensity or more, over-expression) or under 0.5 (half intensity or less, under-expression) (0.5 > FC > 2) among treated and untreated samples were selected. Such comparison resulted in two lists of genes differentially expressed in either LB24 melanoma cells [1,173 probes (719 genes, excluding redundancies); 6.2% of the 18,798 analysed ones] (Additional file 1-A) and in BJ fibroblast [1,883 probes (1,177 genes, excluding redundancies); 10%] (Additional file 2-A). In particular, 3.6% and 2.6% analysed transcripts were over- and under-expressed in melanoma cells, respectively. In fibroblasts, the trend of percentages was instead opposite (5.2% under- and 4.8% over-expressed transcripts) (Table 1).

**Table 1:** Transcriptional changes induced by D6 treatment in melanoma cells and normal fibroblasts

**Cell type**	**Differentially expressed transcripts**	**Over-expressed**	**Under-expressed**
	(**0**.**5** >**FC** >**2**)	(**FC** >**2**)	(**FC** <**0**.**5**)
**LB 24 DAGI** Melanoma cells	1173 (6.2%)	681 (3.6%)	492 (2.6%)
**BJ** Fibroblasts	1883 (10%)	910 (4.8%)	973 (5.2%)

Results from the statistical analysis conducted on microarrays gene expression data from LB24 melanoma cells and BJ fibroblasts, treated or not with 10 μM D6, are reported. Data shown represent the number / (percentages) of human transcripts that resulted differentially expressed (0.5 > FC > 2) by comparing treated and not treated samples. Detailed gene expression profile and statistical analysis procedures are described in the Methods section.

The two lists of selected probes were analysed by the Ingenuity Pathway Analysis (IPA) software. Results obtained on melanoma cells are reported in Additional file 1-B and 1-C. IPA results obtained for BJ fibroblasts are reported in Additional file 2-B and 2-C.

### D6 treatment affects cell death and proliferation bio-functions

The top bio-functional categories identified by IPA among the genes modulated in treated melanoma cells are listed in Table 2, where the *p*-*values* range and number of molecules involved are reported for each category (see the complete list in Additional file 1-B).

**Table 2:** Melanoma top bio functions

**Bio-Function**	***p-value***	**N. of molecules**
**1** Cell death	3.34E-15 – 3.24E-02	194
**2** Cellular function and maintenance	6.17E-14 – 3.24E-02	64
**3** Cancer	1.22E-13 – 3.24E-02	306
**4** Cell cycle	9.64E-12 – 3.15E-02	113
**5** Cellular growth and proliferation	1.16E-11 – 3.15E-02	204

Top five bio-functional categories identified by IPA software by analysing the 1,173 transcripts modulated in 10 μM D6 treated LB24 melanoma cells are reported. The *p*-*value* reflects the significance of the enrichment of input genes in each functional category. The complete list is reported in Additional File 1-B.

The lowest *p*-*values* were found for the *Cell Death* category with 194 molecules involved (Table 2, n. 1). Cell death is indeed the primary effect detected on melanoma cells after D6 treatment [21]. Moreover, a variable number of molecules differentially modulated by D6 involved functional categories strictly correlated with cell proliferation processes such as cellular function and maintenance, cell cycle and cell growth and proliferation (Table 2, n. 2–5).

### D6 induces stress response pathways and down-regulates cell proliferation pathways

Table 3 lists the most significant pathways (*p* < 0.05) that IPA found to be enriched with the input genes in melanoma cells (a complete list is reported in Additional file 1-C). For each pathway, the respective nominal *p*-*value*, along with all the input molecules are reported (Table 3).

**Table 3:** Melanoma top canonical pathways

**Ingenuity canonical pathways**	**Nominal *p-value***	**Genes**
**1** Aldosterone Signaling in Epithelial Cells	2.81838E-06	**CRYAB,HSPA7**,SGK1,**DNAJB4,HSPA1A/HSPA1B,HSPH1**,SLC12A2,**HSPA6, DNAJC3,HSPD1,DNAJB2,DNAJB9,HSPA5,DNAJA1,DNAJB14,HSPA4,HSPA1L**,DUSP1,**HSPA13**,**PIK3R2**,**DNAJB6,DNAJB1,HSPA4L**
**2** Protein Ubiquitination Pathway	3.98107E-06	**CRYAB**,UBE2H,**HSPA7**,**DNAJB4,HSPA1A/HSPA1B,HSPA6,DNAJC3,DNAJB2, HSPA5,DNAJA1,HSPA4,HSPA1L**,STUB1,HLA-B, **DNAJB1**, BRCA1, **HSPA4L**, MED20, **HSPH1**, PSMC4, USP30, **HSPD1, DNAJB9, DNAJB14**, SKP2 (includes EG:27401), ANAPC4, PSMD12, **HSPA13, DNAJB6**, UBC, UBE2D3
**3** NRF2-mediated Oxidative Stress Response	7.58578E-05	NRAS,**DNAJB4**,HERPUD1,**DNAJC3**,**DNAJB2**,**DNAJA1**,**DNAJB9**,**DNAJB14**,MAFF, TXNRD1, FOS, HMOX1, JUN, ATF4, MAP2K3, FOSL1, **PIK3R2**, SQSTM1, **DNAJB6**, **DNAJB1**, GCLM, EIF2AK3
**4** Cell Cycle: G2/M DNA Damage Checkpoint Regulation	0.000177828	**CDC25B**, **GADD45A**, **CDKN1A**, TOP2A, **CCNB2**, PKMYT1, BRCA1, SKP2 (includes EG:27401), **CCNB1**
**5** p53 Signaling	0.000194984	**PMAIP1**, **GADD45B**, THBS1, TNFRSF10B, **TP53BP2**, SERPINE2, SCO2 (includes EG:606683), JUN, **GADD45A**, **CDK4**, ADCK3, **CDKN1A**, **PIK3R2**, BRCA1
**6** VDR/RXR Activation	0.000446684	GTF2B,LRP5, **GADD45A**, MXD1, HOXA10, **CDKN1A**, IGFBP3, CD14, CST6 (includes EG:1474), CSF2, RXRA, KLF4
**7** Pyrimidine Metabolism	0.001	POLE2, DCTPP1, UPP1, POLQ, DKC1, DTYMK, RFC5, POLRMT, PUS1, TXNRD1, POLR1C, POLD3, PNP, POLA2, UNG, ENTPD7
**8** Mitotic Roles of Polo-Like Kinase	0.002089296	**CDC25B**, PPP2CB, ANAPC4, PLK2, CDC7 (includes EG:12545), **CCNB2**, PKMYT1, ANAPC13, **CCNB1**
**9** IGF-1 Signaling	0.002818383	SOCS1, CSNK2A2, FOS, JUN, NRAS, CTGF, NOV, IGFBP3, PRKAG2, IRS2, **PIK3R2**, CYR61
**10** Hereditary Breast Cancer Signaling	0.003467369	NRAS, **GADD45B,** FANCG, RFC5, **CCNB1**, PALB2, **GADD45A**, **CDK4**, XPC, **CDKN1A**, **PIK3R2**, UBC, BRCA1
**11** ATM Signaling	0.003890451	JUN, **GADD45B**, **GADD45A**, **CDKN1A**, **CCNB2**, ATF4, BRCA1, **CCNB1**
**12** RAR Activation	0.004265795	RDH14, BMP2 ,CSK, ADCY3, SMAD5, **NFKB1**, VEGFA, FOS, CSNK2A2, JUN, TAF4 (includes G:100149942), DUSP1, CRABP2, IGFBP3, PRKAG2, **PIK3R2**, RXRA
**13** Cyclins and Cell Cycle Regulation	0.004677351	PPP2CB, E2F6, TFDP1, **CDK4**, SUV39H1, **CDKN1A**, E2F5, **CCNB2**, SKP2 (includes EG:27401), **CCNB1**
**14** p38 MAPK Signaling	0.005495409	FADD, **DDIT3**, DUSP1, DUSP10, MEF2D, H3F3A/H3F3B, ATF4, MAP2K3, MKNK2, EEF2K, H3F3C, IRAK2
**15** Pancreatic Adenocarcinoma Signaling	0.005888437	VEGFA, HMOX1, E2F6, TFDP1, **CDK4**, SUV39H1, **CDKN1A**, E2F5, HBEGF, **PIK3R2**, ERBB2, **NFKB1**
**16** Renal Cell Carcinoma Signaling	0.00691831	VEGFA,ETS1,FOS,JUN,NRAS,SLC2A1,CRK,**PIK3R2**,UBC
**17** Cell Cycle Regulation by BTG Family Proteins	0.00691831	PPP2CB,E2F6,**CDK4**,E2F5,BTG1,CCRN4L
**18** Retinoic acid Mediated Apoptosis Signaling	0.007585776	FADD,TNKS,CRABP2,TNFRSF10B,TNFRSF10D,RXRA,TIPARP,IRF1 (includes EG:16362)
**19** Glucocorticoid Receptor Signaling	0.008912509	IL8,NRAS,**HSPA7**,SGK1,**HSPA1A/HSPA1B**,**HSPA6**,**NFKB1**,**HSPA5**,TAF13 (includes EG:310784),FOS,**HSPA4**,GTF2B,TAF6L,JUN,TAF4 (includes EG:100149942),**HSPA1L**,DUSP1,**CDKN1A**,FKBP4,PRKAG2,**PIK3R2**,CSF2
**20** Toll-like Receptor Signaling	0.01023293	FOS,LY96 (includes EG:17087),JUN,CD14,MAP2K3,**NFKB1**,IRAK2
**21** Endoplasmic Reticulum Stress Pathway	0.010715193	**DNAJC3**,ATF4,**HSPA5**,EIF2AK3
**22** Neurotrophin/TRK Signaling	0.013489629	FOS,JUN,NRAS,SPRY1,ATF4,MAP2K3,**PIK3R2**,FRS2
**23** Molecular Mechanisms of Cancer	0.015488166	BMP4,SUV39H1,BMP2,CRK,SMAD5,**NFKB1**,**CDC25B**,E2F6,JUN,E2F5,**PIK3R2**, BRCA1,LRP5,PMAIP1,NRAS,STK36,TFDP1,ADCY3,AURKA,FADD,FOS,RND3, **CDK4**,**CDKN1A**,PRKAG2,MAP2K3
**24** Chronic Myeloid Leukemia Signaling	0.015848932	E2F6,NRAS,TFDP1,**CDK4**,SUV39H1,**CDKN1A**,E2F5,CRK,**PIK3R2**,**NFKB1**
**25** Cell Cycle: G1/S Checkpoint Regulation	0.016982437	E2F6,TFDP1,**CDK4**,SUV39H1,**CDKN1A**,E2F5,SKP2 (includes EG:27401)
**26** Role of BRCA1 in DNA Damage Response	0.018620871	E2F6,GADD45A,FANCG,**CDKN1A**,E2F5,RFC5,BRCA1
**27** Role of CHK Proteins in Cell Cycle Checkpoint Control	0.022387211	E2F6,**CDKN1A**,E2F5,RFC5,BRCA1
**28** Bladder Cancer Signaling	0.028840315	VEGFA,IL8,NRAS,TFDP1,THBS1,**CDK4**,SUV39H1,**CDKN1A**,ERBB2
**29** IL-17A Signaling in Gastric Cells	0.033113112	IL8,FOS,JUN,**NFKB1**
**30** Role of Tissue Factor in Cancer	0.036307805	VEGFA,IL8,NRAS,CTGF,ARRB1,PLAUR,HBEGF,**PIK3R2**,CSF2,CYR61
**31** HMGB1 Signaling	0.038904514	IL8,FOS,JUN,NRAS,RND3,MAP2K3,**PIK3R2**,**NFKB1**,KAT2A
**32** ERK/MAPK Signaling	0.039810717	ETS1,NRAS,H3F3A/H3F3B,CRK,MKNK2,PPP1R14B,H3F3C,FOS,PPP2CB,ELF3,DUSP1,PRKAG2,ATF4,DUSP4,**PIK3R2**
**33** Non-Small Cell Lung Cancer Signaling	0.041686938	NRAS,TFDP1,**CDK4**,SUV39H1,**PIK3R2**,ERBB2,RXRA
**34** Prostate Cancer Signaling	0.041686938	NRAS,TFDP1,SUV39H1,**CDKN1A**,NKX3-1,ATF4,**PIK3R2**,**NFKB1**
**35** RAN Signaling	0.042657952	CSE1L,XPO1,RANBP1
**36** Cell Cycle Control of Chromosomal Replication	0.042657952	MCM3,**CDK4**,ORC5 (includes EG:26429),CDC7 (includes EG:12545)
**37** IL-6 Signaling	0.043651583	IL8,SOCS1,CSNK2A2,FOS,JUN,NRAS,CD14,MAP2K3,**NFKB1**
**38** IL-2 Signaling	0.046773514	SOCS1,CSNK2A2,FOS,JUN,NRAS,**PIK3R2**
**39** MIF Regulation of Innate Immunity	0.046773514	FOS,LY96 (includes EG:17087),JUN,CD14,**NFKB1**
**40** Role of IL-17F in Allergic Inflammatory Airway Diseases	0.046773514	IL8,ATF4,SIK1,CSF2,**NFKB1**
**41** Erythropoietin Signaling	0.047863009	SOCS1,FOS,PTPN6,JUN,NRAS,**PIK3R2**,**NFKB1**
**42** Estrogen Receptor Signaling	0.048977882	TAF6L,GTF2B,NRAS,TAF4 (includes EG:100149942), MED20, H3F3A/H3F3B, MED16,HNRNPD,TAF13 (includes EG:310784),MED10,H3F3C

Most significant pathways (nominal *p*-*value* < 0.05) identified by IPA software by analysing the 1,173 transcripts modulated in 10 μM D6 treated LB24 melanoma cells are reported. The *p*-*value* reflects the significance of the enrichment of input genes in each pathway. The complete list is reported in Additional File 1-C. Genes discussed in the text and HSPs are evidenced by bold characters.

**Figure 3:**
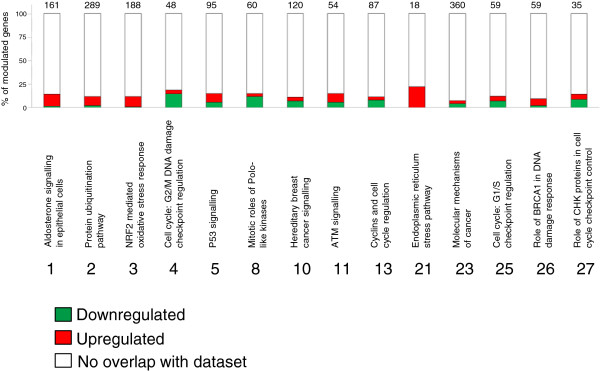
**Melanoma significant pathways.** Diagram of most representative pathways modulated by D6 treatment in melanoma cells. X axes: representative molecular pathways. Pathways are numbered according to Table 3; Y axes: percentage of D6 modulated genes for each pathway. Numbers on top of the columns represent the total number of genes involved in each pathway.

A general trend of up-regulation for pathways involving cell stress response was evident (Figure 3, red bars); conversely, pathways that control cell proliferation appeared down-regulated (Figure 3, green bars).

The first three most significant pathways, 1-*Aldosterone signalling*, 2-*Protein ubiquitination*, and 3-*NRF2 mediated oxidative stress response* as well as the 21-*Endoplasmic reticulum stress* pathway appear to be up-regulated (Table 3; Figure 3), depicting a strong activation of stress induced molecular responses that involves over-expression of heat shock proteins (HSPs) and activation of protein degradation processes. Among up-regulated HSPs, *HSPA6* is the most over-expressed transcript in melanoma treated cells with a FC value of 368.61 (Table 4). *HSPA6* codifies for the Hsp70B’, a highly stress inducible protein.

**Table 4:** Melanoma modulated genes

**GENE**	**FC**	
	**LB**	**BJ**
**HSPA6**	368.61	480.00
**HSPA1A**	20.35	12.38
**CDKN1A**	19.35	4.29
**DDIT3**	11.11	9.11
**GADD45A**	7.78	6.00
**GADD45B**	7.47	14.90
**PMAIP1**	6.08	7.72
**BCL10**	3.93	2.02
**TP53BP2**	2.36	–
**CCNF**	0.24	–
**KIT**	0.30	–
**CDK4**	0.43	0.43
**CCNB1**	0.43	–
**CDC25B**	0.47	–
**NFKB1**	0.47	–
**PIK3R2**	0.48	–

Genes differentially expressed in LB24 melanoma cells after D6 treatment are reported. The most significant genes possibly related to D6 antiproliferative and pro-apoptotic effects as well as their relative fold change values (FC) in either LB24 melanoma cells or BJ fibroblasts are presented. The complete lists of differentially expressed genes and relative FC for each cell line are reported in the Additional files 1-A and 2-A respectively.

Additional pathways such as 4 -*Cell cycle:G2*/*M DNA damage checkpoint regulation*, 5 -*p53 signalling*, 10 -*Hereditary breast cancer signalling*, 11 -*ATM signalling*, 26 -*Role of BRCA1 in DNA damage response*, and 27 -*Role of CHK proteins in cell cycle checkpoint control* are all related to DNA repair mechanisms and cell death triggering (Table 3 and Additional files 3, 4, 5, 6, 7, 8), evidencing a DNA damage as cell response to D6 treatment.

The up-regulation of pathway 5- *p53 signalling* (Figure 3, Additional file 4), which acts in response to cell injury or DNA damage by controlling cell proliferation and driving cells to apoptosis, is noteworthy and points out a central role of this regulatory protein in the D6 anticancer effect on melanoma cells. Indeed, induction of p53 activity was evidenced by up-regulation of some p53 target genes: *CDKN1A* (FC = 19.35), *GADD45A* and *B* (FC = 7.78 and FC = 7.47, respectively), all codifying for inhibitors of the cell cycle, and *PMAIP1* (FC = 6.08) codifying for Noxa, a pro-apoptotic BH3 only protein of the Bcl2 family (Table 4). A slight up-regulation of *TP53BP2* gene (FC = 2.36), codifying for ASSP2 - a member of the ASPP (apoptosis-stimulating protein of p53) family of p53 interacting proteins, confirms the role of p53 as an apoptosis activator in our system.

Down-regulation of pathways controlling cell proliferation such as pathways 4-*Cell cycle*:*G2*/*M DNA damage checkpoint regulation*, 8 -*Mitotic roles of PLK*, 13 -*Cyclins and cell cycle regulation*, 23 -*Molecular mechanisms of cancer* and 25 -*Cell cycle*: *G1*/*S checkpoint regulation* was also observed (Figure 3, green bars). These alterations seem to be related to the down-regulation of important cell-cycle “motors” like *CCNB1*/*2* (cyclin B1 and 2), *CDC25*, and *CDK4* (Tables 3 and 4, Additional files 3, 9, 10, 11, 12).

Other interesting features highlighted by the pathway analysis are the partial down regulation of phosphatidylinositol 3 kinase - regulatory subunit 2 (*PIK3R2*, FC = 0.48) and nuclear factor-kB1 (*NFKB1*, FC = 0.47) genes (Table 4). On this regard, both *PIK3R2* and *NFKB1* are greatly represented in the melanoma most significant pathways listed in Table 3 (19 and 13 out of 42 pathways, respectively).

### D6 modulates the expression of several life and death regulator genes

Beside the modulated genes highlighted by the pathway analysis, FC analysis evidenced some other genes whose modulation might have a role in the observed antiproliferative and pro-apoptotic activities of D6 on melanoma cells (Additional file 1-A) [21]. Among them, the *CCNF* gene that codifies for the G2/mitotic-specific cyclin-F was highly down-modulated (FC = 0.24, Table 4). Cyclin F is produced in the G2 cell cycle phase and is essential for fidelity of mitosis; therefore, a down-modulation of such protein may interfere with the progression of cell division, which is consistent with the block of cell cycle in G2/M phase observed upon D6 treatment (Figure 2).

Among the down-regulated genes (FC < 0.5), we found an evident under-expression of the *c*-*KIT* proto-oncogene (FC = 0.30), whose activation is often associated with increased cell proliferation, specifically in melanoma (Table 4) [24]. Down regulation of *c*-*KIT* is then likely to be related to D6 anticancer activity on melanoma cells, contributing to inhibit cell-proliferation signals.

We already demonstrated that D6 treatment induces apoptosis in melanoma cells through the mitochondrial intrinsic pathway [21]. Looking at gene expression levels of the apoptosis-related genes, we observed a strong up-regulation of *DDIT3* (DNA–damage-inducible transcript 3, FC = 11.1), a transcription factor activated in endoplasmic reticulum stress conditions that promotes apoptosis by induction of caspases [25], as well as a discrete over-expression of the gene *BCL10* (FC = 3.93), encoding for a pro-apoptotic member of the Bcl2 family proteins [26], in addition to the over-expression of the protein Noxa codified by *PMAIP1* mentioned above (FC = 6.08) (Table 4). These are further evidences about the involvement of pro-apoptotic signals in D6 treated cells.

### Expression profile changes in D6 treated fibroblasts

The IPA-based analysis of the 1,883 transcripts modulated by D6 in fibroblasts (listed in Additional file 2-A) was useful to compare results with those obtained in melanoma cells. Biological function categories found to be significant in fibroblasts were similar to those selected for melanoma cells (Additional file 2-B), suggesting that D6 treatments involve life and death controlling mechanisms also in normal cells. However, D6 treated fibroblasts did not show significant effects in terms of block of proliferation or induction of apoptosis, as we previously described [21]. The analysis of D6 treated fibroblasts evidenced the involvement of the pathways underlying general cell stress responses (Additional file 2-C). However, processes including chaperones activation and protein degradation were less significant in fibroblasts than in melanoma cells (Additional file 2-C, pathways 10, 16, 21), with some HSPs being down-modulated. Conversely, DNA damage induced cell response pathways were highly significant in fibroblasts also (see Additional file 2-C, pathways 2, 3, 4, 5, 9, 18), indicating that D6 even triggers an anti-mitotic reaction in normal cells (though not confirmed in the biological assays [21]). Such a response was anyway weaker in these latter cells and pathway trends markedly differed between melanoma and fibroblasts (i.e., p21/*CDKN1A*, FC = 19.35 in melanoma cells versus FC = 4.29 in fibroblasts, or *CCNB1*, FC = 0.43 and *CDC25B*, FC = 0.47 in melanoma cells with no modulation in fibroblasts; Table 4). Furthermore, neither *PIK3R2* nor *NFKB1* gene expressions were altered in fibroblasts, suggesting that the relative pathways are not hindered by D6 in these normal cells. These data suggest that D6 interaction with both PI3K/Akt and NF-kB signal transduction cascades may be peculiar of its activity on cancer cells.

### Protein levels reflect gene expression changes in D6 treated melanoma cells

**Figure 4:**
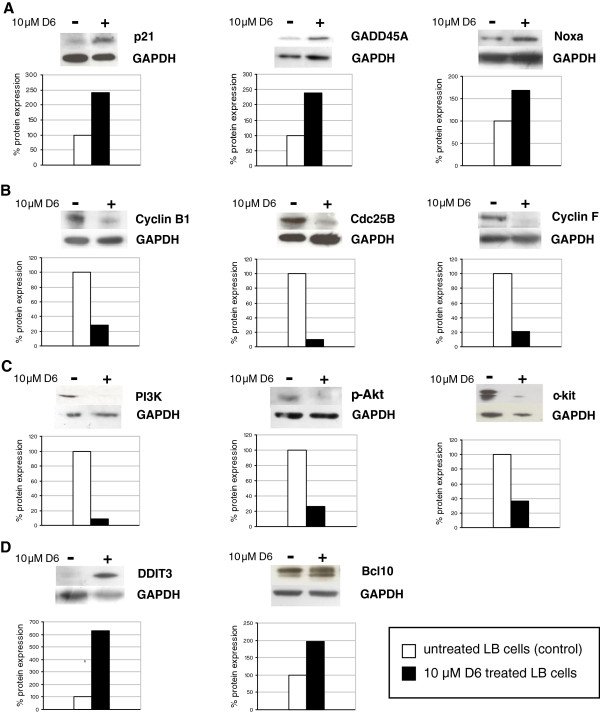
**Western blot analysis.** Validation by western blotting of selected gene products expression. LB24 melanoma cells were grown to semi-confluence, treated (+) or not (−) for 24 hours with 10 μM D6, then harvested and lysed as described in Methods. Cell lysates were analysed by western blotting for the expression levels of selected proteins modulated by D6 treatment: (**A**) *p53* targets: *p21*, *GADD45A* and *Noxa*; (**B**) cell cycle regulators: *cyclin B*, *cdc25*, *cyclin F*; (**C**) growth signals stimulating proteins: *PI3K*, *p*-*Akt*, *c*-*kit*; (**D**) proapoptotic proteins: *DDIT3*, *Bcl10*. Protein levels were quantified by Image J Software and normalized over (ratio) the GAPDH protein levels used as internal control. Data are reported as percentage of protein expression in D6 treated cells compared to untreated ones (100%).

Protein levels for most of the differentially expressed genes above mentioned were verified by western blot on LB24 cells (Figure 4), in order to confirm that D6-induced modulation of expression at mRNA levels was indeed maintained at protein levels.

Figure 4A shows the increased protein levels detected by western blot for the three major p53 targets modulated by D6: p21, GADD45A, and Noxa. The p21 protein was about 2.5 fold more expressed in treated cells compared to the untreated ones, confirming the increase of *CDKN1A* gene expression. Same increased levels were observed for the GADD45-A protein, while Noxa protein levels were about 70% higher as compared to those of control cells.

Among the proteins involved in regulation of cell cycle G2/M phase transition, we performed immunoblot assays for cyclin B, cdc25, and cyclin F. Protein levels detected in D6 treated cells were much lower than those of untreated ones, thus confirming the decrease of expression observed in microarrays analysis (Figure 4B).

An almost complete depletion of the PI3K protein in treated cells compared to untreated ones is shown in Figure 4C, reflecting the under-expression of the *PIK3R2* gene and suggesting a possible down-regulation of PI3K/Akt pathway. To confirm such an inhibitory effect, we investigated the Akt activation status and performed an immunoblot analysis using a specific anti phospho-Akt antibody. Expression of *AKT* gene itself was not modulated after D6 treatment (data not shown), but its phosphorylation/activation status was decreased of about 75% (Figure 4C). Down-modulation of *c*-*KIT* gene expression was also confirmed by western blot analysis, which showed that c-kit protein level was decreased of about 65% (Figure 4C).

Finally, an up-modulation of the DDIT3 and Bcl10 protein expression levels upon D6 treatment was confirmed by western blot analysis (Figure 4D).

## Discussion

In this paper, we demonstrate that the curcumin analogue D6 is able to enter melanoma cells reaching a peak in about two hours (see Figure 1), and give rise to a series of molecular changes that underlie the previously described anti-tumour activity of such a compound [21].

Our results indicated that D6 treatment may promote a block of cell cycle progression in G2 phase (see Figure 2) and this could represent one of the mechanisms that inhibit melanoma cells growth, as previously observed [21]. Alterations in cell cycle progression are indeed important events in cancer development and hindering such altered mechanism has been often used as a good strategy to inhibit tumour growth [27].

To investigate the possible molecular mechanisms triggered by D6 treatments, we undertook a gene expression profile analysis on melanoma cancer cells and fibroblast normal cells. Our primary objective was to identify genes that were up- or down-regulated in response to treatment, and which could be related to the phenotypic outcome. Two lists of regulated transcripts (0.5 > FC > 2), one for LB24 melanoma cells and the other for BJ fibroblasts were selected (Additional file 1-A and 2-A, respectively) and subsequently analyzed by the IPA software. Considering both the most significant functional categories and canonical pathways (see Tables 2 and 3, respectively), the activity of D6 compound in melanoma cells is certainly based on either cell stress responses either activation/repression of mechanisms regulating cell survival. Pathway analysis revealed up-regulated effectors of cell stress response and protein degradation as well as down-regulated gene products controlling cell proliferation (see Figure 3). The activation of cell defence pathways observed on melanoma cells (Table 3, pathways 1–3) indicates that D6 treatment causes important stimulation of the cellular stress response, with a strong induction of HSPs, which in turn affects cell survival and drives toward cell death [28-30]. In physiological or pathological conditions, cellular stress leads to transport and accumulation of damaged proteins in the endoplasmic reticulum where they should be repaired or committed to degradation. This stimulates the over-expression of chaperons and HSPs that perform a sort of quality control and drive seriously damaged proteins to ubiquitination and proteasome degradation. When endoplasmic reticulum functions are strongly compromised, this organelle triggers apoptotic signals in order to eliminate the irreversibly damaged cell [31]. In our model, several HSPs genes show to be up-regulated (see Table 3), and *HSPA6* is the most over-expressed transcript (FC = 368.61, Table 4). It codifies for the stress inducible Hsp70B’ protein, normally under-expressed or absent in most cell types, whose expression is strictly linked to that of Hsp72 (gene *HSPA1A*, FC = 20.35, Table 4); both these proteins have a key role in mediating cell survival during endoplasmic reticulum proteotoxic stress conditions [32]. One could speculate that their huge increase of expression levels following D6 treatment could be related to the extreme endoplasmic reticulum stress response that finally directs melanoma cells to death by triggering apoptosis. In support of this hypothesis, our analysis evidenced a strong induction of the *DDIT3* gene (FC = 11.1, Table 4), also known as growth arrest and DNA damage-inducible gene 153 (*GADD153*), which is one component of the ER stress-mediated apoptosis pathway [25]. Increased expression of *GADD153* by curcumin has been previously reported as one of the first steps toward apoptosis in colon cancer cells [33], thus confirming a possible contribution in this sense in D6 treated melanoma cells also.

Analogously, D6 stimulated cell stress response pathways in fibroblasts, but at a lower level compared to melanoma cells (Additional file 2-C). This is confirmed by the over-expression of single genes - like several *HSPs* and *DDIT3* (Table 4), which is presumably milder or not sufficient to promote cell death [21].

One noteworthy feature occurring in melanoma cells upon D6 treatment - as evidenced by IPA - is an up-regulation of the p53 signalling pathway (Figure 3). The p53 tumour suppressor protein is a key transcriptional regulator that responds to a variety of cellular stresses and controls key cellular processes such as DNA repair, cell-cycle progression, angiogenesis, and apoptosis. The p53 protein thus acts like a “driver”, which can either save injured cell by inducing damaged DNA repair and let it to re-enter cycle or sacrifice it by stimulating both cell cycle arrest and apoptosis [34,35]. In our model, up-regulation of p53 signalling pathways seems to have a key role in mediating both antiproliferative and pro-apoptotic effects of D6 on LB24 melanoma cells (see Additional file 4). Indeed, a strong up-regulation of some p53 target genes has been detected and could explain the anticancer effects of D6: *CDKN1A* and *GADD45A*/*B* - that are strong inhibitors of cell cycle G2/M transitions [36,37], might be responsible for the block of cell cycle at G2 phase, and Noxa - a pro-apoptotic BH3-on*ly* protein of the Bcl-2 family, may account for the apoptotic cell death [38]. As a confirmation of this, the expression of *CDKN1A* gene codifying for the CDK inhibitor p21 is about 20 times higher in treated melanoma cells (FC = 19.35, Table 4). The p21 protein belongs to the Cip/Kip family of inhibitors and inactivates CDK-cyclin complexes [36,39]. In our system, it seems to regulate large part of melanoma cells response to D6 compound, being a component of most pathways identified by IPA (Table 3, pathways 4, 5, 6, 10, 11, 13, 15, 19, and 23; see also Additional files 3, 4, 5, 6, 7, 8, 10, 11, 12). The strong up-regulation of *GADD45A* and *B* (respectively FC = 7.78 and FC = 7.47; see Table 4) also appears to influence several growth controlling pathways (Table 3, pathways 4, 5, 6, 10, 11, and 26; see Additional files 3, 4, 5, 6, 7). Proteins encoded by these two stress induced genes are involved in regulation of growth and apoptosis and may cooperate in inhibiting cell growth [37,40]. Over-expression of the BH3-only Noxa protein codified by the *PMAIP1* gene (FC = 6.08, Table 4) suggests that D6 induced apoptosis could be partially p53 dependent. Noxa and Puma (p53-upregulated modulator of apoptosis) proteins are in fact direct targets in p53-mediated apoptosis at mitochondrial level [38,41], functioning as “sensors” for apoptotic signals. Thus, increased Noxa levels could participate in initiating the apoptotic cascade in D6 treated melanoma cells. Supporting this hypothesis, a slight up-regulation of the *TP53BP2* gene has been reported in our melanoma model (FC = 2.36, Table 4). This gene encodes for ASPP2, a member of the ASPP (apoptosis-stimulating protein of p53) family of p53 interacting proteins, which regulate the apoptotic function of p53 and its family members, p63 and p73. Biochemical and genetic evidences have shown that ASPP1 and ASPP2 activate the apoptotic but not the cell-cycle arrest function of p53 [42]. The increased levels of ASPP2 protein observed in D6 treated melanoma cells might thus induce p53 to trans-activate its pro-apoptotic target genes, resulting in the observed over-expression of Noxa, and subsequent activation of mitochondrial intrinsic apoptosis. Another evidence of pro-apoptotic signals in D6 treated cells expression profile is the over-expression of the *BCL10* gene (FC = 3.93, Table 4), encoding for a pro-apoptotic member of the Bcl2 family proteins. Bcl10 protein contains a caspase recruitment domain (CARD) motif and promotes the activation of caspase-9 [26].

The p53 signalling pathway has resulted to be significantly affected also in fibroblasts (Additional file 2-C), being *CDKN1A* and *GADD45 A*/*B* partially up-regulated. Again, this molecular response in fibroblasts is weaker than that in melanoma cells (*CDKN1A* FC = 4.29, Table 4), without causing in normal cells block of proliferation or cell death.

Our analyses pointed out a down modulation of cell cycle regulators cyclin B1, cdc25B, and CDK4 (Table 4), which certainly contributes to the inhibition of cell proliferation exerted by D6 on melanoma cells. Block of cell cycle in G2/M phase perfectly matches with a decrease in expression of both cyclin B (the mitotic switching cyclin) and cdc25 (the CDK1 activating phosphatase), whereas the decrease in CDK4 expression indicates that cells lack entering the cell cycle while are driven to age and die [39], as demonstrated by the G1 cell population decrease after D6 treatment (see Figure 2). Interestingly, a lower or absent down-modulation of these mitosis promoters has been evidenced in fibroblasts (Table 4), suggesting that D6 treatment specifically inhibits cell proliferation pathways in melanoma cells.

Another gene down-modulated by D6 in melanoma cells (and not in fibroblasts) is the *CCNF* gene (FC = 0.24, Table 4), codifying for cyclin F, the founding member of the F-box protein family [43]. In addition to an F-box domain, cyclin F contains a cyclin-box domain, but, in contrast to typical cyclins, it does not bind or activate any cyclin-dependent kinases (CDKs). However, like other cyclins, cyclin F protein levels vary during the cell division cycle, peaking in G2. During G2, cyclin F is involved in ubiquitination and degradation of proteins [44] as well as in spindle formation and it is required for the fidelity of mitosis and genome [45]. In our system, down-modulation of such a protein is in agreement with the block of cell cycle in G2/M phase demonstrated by cytofluorimetry (Figure 2).

A further contribution to D6 anticancer activity on melanoma cells is given by the down-modulation of the *c*-*KIT* proto-oncogene (FC = 0.30, Table 4). The c-kit protein belongs to class III receptor tyrosine kinases; its extracellular domain binds the SCF (stem cells factor) to stimulate several processes, including melanogenesis, gametogenesis, and haematopoiesis [46]. The *c*-*KIT* up-regulation is often associated with increased cell proliferation; its down-regulation in D6 treated melanoma cells was confirmed by western blot analysis (Figure 4C).

One could also hypothesize that a big contribution to the anticancer activity of D6 is given by down-regulation of both phosphatidylinositol 3-kinase (PI3K) and NF-kB signalling pathways. There is growing evidence that activation of the *PI3K*/*Akt* pathway plays a significant role in melanoma (reviewed in [47]). Our results are consistent with an inhibition of PI3K/Akt pathway activation in melanoma cells following D6 treatment. As also confirmed by western blot analysis, a decreased expression of the *PIK3R2* gene (FC = 0.48, Table 4), an almost complete depletion of the PI3K protein, and a 75% decrease of activated phospho-Akt have been observed in D6 treated cells (Figure 4C). In addition, a slight up-modulation of PTEN (Akt antagonist) gene expression was detected in our study (FC = 1.45, data not shown). The IκB kinase (IKK)/NF-κB signalling pathway is also often altered in tumours and NF-κB can affect all six hallmarks of cancer through the transcriptional activation of genes associated with cell proliferation, angiogenesis, metastasis, tumour promotion, inflammation and suppression of apoptosis (reviewed in [48]). PI3K and NF-kB signalling pathways are functionally linked, being NF-kB possibly activated by Akt kinase [49]. Our results show that, similarly to *PIK3R2*, *NFKB1* gene expression is down-regulated by D6 in melanoma cells (FC = 0.47), but it is unclear whether this could be due to the PI3K/Akt signalling repression. Deeper investigations should be made to shed light on this molecular event. However, it is interesting to underline that PI3K and NF-kB pathways are both involved in curcumin anti-tumour activity [50] and inhibition of NF-kB activation may account for curcumin efficacy on cancer cells [18] and, specifically, on human melanoma cells [19,20]. As a consequence, it is likely that the curcumin analogue D6 shares some mechanisms of action with its natural compound, being even more effective in inhibiting tumour cells growth [21]. It is noteworthy that neither *PIK3R2* nor *NFKB1* genes expression was modulated in D6 treated normal fibroblasts (Table 4). Based on these considerations, we can postulate that PI3K and NF-kB signalling down-regulation is strongly related to the anticancer activity of D6 on melanoma cells.

A further consideration may be done about a possible relationship between *NFKB1* under-expression and p53 signalling up-regulation. An intense crosstalk exists among these two transcription factors that activate the expression of genes with opposite functions. They are indeed competitors for the transcriptional coactivator p300/CBP [51] and, depending upon which of them (NF-κB or p53) recruits this protein, different downstream pathways will be activated, resulting in either cell proliferation or growth arrest and apoptosis [52]. To this regard, a recent report by Sen and colleagues [53] demonstrated that curcumin reverses doxorubicin resistance in breast cancer by inhibiting NF-κB activation and thus rescuing p300 coactivator, which in turn becomes available to the p53 transcription factor, and finally allows p53-dependent transactivation of proapoptotic proteins such as Bax, PUMA and Noxa. Based on these observations down-regulation of NF-κB by D6 would make the coactivator p300 available for recruitment by p53, thus favouring transactivation of its target genes that triggers antiproliferative and proapoptotic activity. This could be a very interesting feature of D6 because its potentiality to both inhibit NF-κB and, at the same time, rescue p53 signalling could be exploited either for direct therapeutic interventions against cancer, but also in combined therapies in order to sensitize resistant cancer cells to chemotherapeutic agents that can stimulate apoptosis by inducing DNA damages and triggering p53 apoptotic signals.

**Figure 5:**
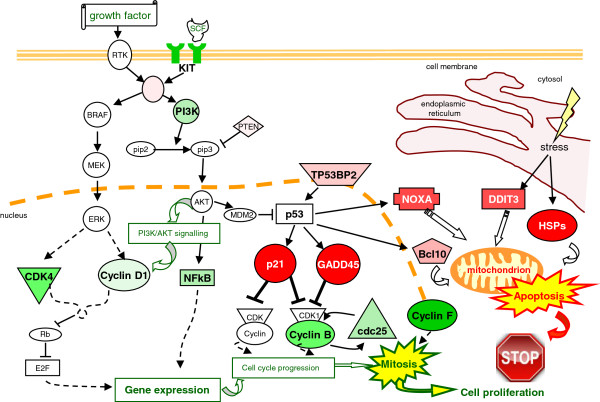
**Summary.** The diagram summarizes the major gene expression changes induced by D6 in melanoma cells, outlining their possible role in determining cell fate. Up-regulated proteins are represented in red gradations, while down-regulated ones are in green. Arrow-head lines indicate molecular activation; blunt-head lines indicate inhibition.

In summary, based on gene expression profile analysis results, we can speculate that different molecular mechanisms may contribute to the anticancer effect of D6 in melanoma cells: i) the induction of a cell stress response that triggers the ER stress-mediated apoptosis pathway; ii) the up-regulation of p53 signalling, which promotes p21- and GADD45-dependent cell cycle arrest as well as mitochondrial apoptosis based on Noxa over-expression; iii) the down-modulation of several growth signals, like both PI3K and NF-kB pathways, and c-kit receptor. The diagram in Figure 5 summarizes the major gene expression changes induced by D6 in melanoma cells and hypothesizes the possible intervention of these changes in depicting cellular fate.

## Conclusions

Altogether, our findings contribute to unveil the molecular mechanisms underlying the anti-tumour activity of D6 in melanoma cells. Based on such results, we can speculate that: a) p53 protein may play a key role in sustaining the anticancer effects exerted by D6 on melanoma cells; b) induction of strong cell stress responses may contribute to the reinforcement of the proapoptotic trend of p53 signalling; and c) down-modulation of several growth signals (c-kit, PI3K/Akt and NF-kB), as well as the under-expression of cell cycle regulators (cyclin B, cdc25 and CDK4) might be involved in cell growth inhibition. This last aspect seems to be peculiar of the response to D6 treatment in melanoma cells, being absent in D6-treated fibroblasts expression profile.

Although our analyses were not exhaustive, data here presented strongly indicate that a huge amount of molecular changes does participate in determining the molecular mechanism of action of D6 on melanoma cells. Gene expression profile analyses on additional melanoma cell lines are currently in progress, in order to either confirm our findings in a larger samples’ collection or evaluate the effects of D6 on both primary and metastatic tumour derived cell lines.

## Methods

### Cell cultures and D6 treatments

Malignant melanoma LB24Dagi (LB24) cell line was obtained from the Department of Molecular and Cellular Biology at the Istituto Dermopatico dell'Immacolata (IDI) in Rome (Italy). Normal human fibroblast BJ (CRL-2522) were purchased from the American Type Culture Collection (ATCC). All cells were grown in RPMI (Invitrogen, Carlsbad, CA, USA) media, supplemented with 10% FBS and penicillin-streptomycin (100 IU/50 μg/ml), as described [54]. The αβ-unsaturated ketone D6 [(*3E*,*3*'*E*)-*4*,*4*'-(*5*,*5*',*6*,*6*'-*tetramethoxy*-[*1*,*1*'-*biphenyl*]-*3*,*3*'-*diyl*)*bis*(*but*-*3*-*en*-*2*-*one*)] (Figure 1A) has been synthesized in our lab as previously described [21]. Stock solution of D6 was prepared by dissolving D6 in DMSO to a final concentration of 100 mM and stored at −20°C. Working solutions of D6 were prepared daily as previously described [21]. Cells were untreated or treated with medium containing 10 μM D6 for different times depending on the experiment, then harvested with 0.25% trypsin-EDTA (Sigma-Aldrich, St. Louis, MO, USA) and processed according to the protocol of the specific analysis they have been submitted.

### D6 cellular uptake

Melanoma cells were plated in T25 tissue culture flasks (8 × 10^5^ cell/flask) in complete medium; after 24 hours cells were treated or untreated with 10 μM D6 for 1, 2, 4, 6 or 24 hours. At each time, cells were harvested with 0.25% trypsin-EDTA solution (Sigma-Aldrich, St. Louis, MO, USA), washed and resuspended in methanol (1 ml). To achieve D6 extraction, cells in methanol were sonicated for 15 min and the cell lysates were centrifuged at 10,000 rpm for 5 min [55]. The supernatants were transferred and stored at −20°C pending analysis. Immediately prior to analysis, the samples were warmed up to room temperature. After vortexing and centrifugation, 100 μl of the sample were filtered and transferred to a HPLC vial for LC/MS analysis.

### LC/MS analysis

LC-grade methanol, acetonitrile, and acetic acid (AA) were purchased from Mallinckrodt J.T. Baker (Deventer, Holland). Water was purified by a Milli-Q Academic System from Millipore (Bedford, MA, USA). Syringe filters (Surfactant Free Cellulose Acetate 0.2 μm 0.13 mm) were purchased from Nalgene Company (Rochester, NY, USA).

Stock solutions of D6 were prepared by dissolving 5 mg of D6 in 10 mL of DMSO (final concentration 500 μg/mL). Stock solutions of D6 were stored at - 20°C in high-density polypropylene cryogenic-vials. Working solution of D6 was prepared daily at the concentration of 100 nM by diluting an aliquot of the stock solutions with the solvent system (Eluent A) and was used to spike samples. Standard curves solutions of D6 at six different concentrations (6.25, 12.50, 25, 50 or 500 nM.) were obtained by adding appropriate concentrations of working solution in samples and solvent system. A 1100 series LC/MSD system (Agilent Technologies, Palo Alto, CA, USA) equipped with a diode-array detector and an autosampler (G1313A) was used for LC separation. Chromatographic separation was achieved using a Polar Plus column (150 mm×2.1 mm, 3 m) (Phenomenex, Torrance, CA, USA) fitted with a 3 μ Polar Plus security guard cartridge (4 mm×2.1 mm) (Phenomenex, Torrance, CA, USA). The column temperature was maintained at 35°C. The mobile phase consisted of Eluent A water with 0.1% HOAc and Eluent B acetonitrile. The separation was performed in a run time of 20 min under gradient conditions with a flow rate of 0.3 mL/min and was followed by clean-up and equilibration stage. The gradient elution ranged from 35% (*t* = 0 min) to 65% acetonitrile (*t* = 20 min). The injection volume was 10 μL. Mass spectrometric detection was performed using an Agilent G1946 (MSD 1100) single stage quadrupole instrument equipped with an electrospray atmospheric pressure ionization source. The system was calibrated with the procedures provided by Agilent; the mass spectrometer was optimized with an infusion of 0.5 μg/mL D6 solution at a flow rate of 100 μL/min. The LC/MS system was programmed to divert column flow to waste for 2.5 min after injection, after which time flow was directed into the mass spectrometer that operated in positive ion mode. For quantitative measurement of analytes, selected ion monitoring (SIM) was employed. In the ESI ion source, D6 formed predominantly the ion at *m*/*z 411* ([M+H]+). The following ESI conditions were applied: drying gas (nitrogen) heated at 350°C at a flow rate of 9.5 L/min; nebulizer gas (nitrogen) at a pressure of 42 psi; capillary voltage in positive mode at 3500V; fragmentor voltage at 70V.

### Cell cycle progression analysis

LB24 cells were plated in 6-well plates (8 × 10^5^ cells/well), let grown overnight and then treated with either 5 μM or 10 μM D6 for 24 hours. After treatments the cells were harvested with trypsin /EDTA and washed with PBS. Pellets were resuspended in 70% cold ethanol and stored at −20°C until analysis. On the day of analysis, ethanol was removed by centrifugation; pellets were washed with PBS and resuspended in 1 ml of PBS containing 50 μg/mL Propidium Iodide, 100 μg/mL ribonuclease and 100 μg/mL sodium citrate (all from Sigma-Aldrich, St. Louis, MO, USA). Samples were then incubated for 30 min at 4°C in the dark and analyzed by flow cytometry using FACS Canto II (BD Biosciences, San Jose, CA, USA). Data analysis was performed using the ModFit LT 3.0 software (Verity Software House).

### Gene expression profile analysis

Total RNA was isolated from LB and BJ cells, untreated or treated with 10 μM D6 for 16 hours, using *AllPrep DNA*/*RNA Mini kit* (Qiagen, Inc., Chatsworth, CA) for a total of 12 RNA samples (4 samples in triplicate: LB-C_1-3_, LB-D6_1-3_, BJ-C_1-3_, BJ-D6_1-3_). The amount of the total RNA was detected using a NanoDrop 2000 (Thermo Fisher Scientific) and the quality was evaluated by agarose gel electrophoresis. The total RNA samples were normalized and, the mRNAs were amplified and labeled using *Illumina*® *TotalPrep*™ *RNA Amplification Kit* (Ambion®). The system uses the *in vitro* transcription (IVT) technology, based on the RNA amplification protocol developed by James Eberwine and coworkers [56]. The first reaction of the IVT is a reverse transcription of mRNAs, performed using an oligo(dT) primer tagged with a phage T7 promoter, and convert the mRNA fraction to single-stranded cDNA. Then, a Second-Strand Synthesis reaction converts the single-stranded cDNA in double-stranded cDNA. This product becomes the template for the in vitro transcription performed using a T7 RNA Polymerase and Biotin-NTP mix. The final results of the three reactions are hundreds to thousands of biotinylated, antisense RNA copies of each mRNA per sample. According to the manufacturer’s protocol, labeled cRNAs were quantified, 750 ng of each sample were denaturated at 65°C for 5 min and hybridized on a HumanHT-12 v3 Expression BeadChip (Illumina®) at 58°C overnight. Each well targets 48,803 human probes, representing the whole pool of human expressed genes. After stringency washing, the signal was developed with streptavidin-Cy3, the array slide was dried by centrifugation and scanned using *iScan System* (Illumina®). Images were processed and signals were quantified and normalized using *GenomeStudio software* (Illumina®). Probes with *detection p*-*value* > 0.05 in more than 9 out of 12 samples were excluded from the statistical analysis.

### Statistical analysis

Statistical analysis was carried out on probes that showed *p*-*values* < 0.05 in 9/12 samples, by using the BRB-Array Tools from Biometric Research Branch of National Cancer Institute – NIH (USA). We identified genes that were differentially expressed as an effect of D6 administration using a random-variance t-test. The random-variance t-test is an improvement over the standard separate t-test as it permits sharing information among genes about within-class variation without assuming that all genes have the same variance [57]. Genes were considered statistically significant if their *p*–*value* was less than 0.001. A stringent significance threshold was used to limit the number of false positive findings. A per gene FDR was also computed using a univariate permutation test. Briefly, class labels of the samples were randomly permuted N times. For each gene, the permutation *p*-*value* is defined as a proportion of permutations for which the *p*-*values* of the univariate test are smaller than the *p*-*value* computed for the original labeling. Data were further filtered by fold change (FC) considering as differentially expressed probes only those showing 0.5 > FC > 2. The results of the tests are reported in the Additional file 1-A (LB24 melanoma cells) and 2-A (BJ fibroblasts), tabulated along with relevant statistics and hyperlink to gene annotations from the NCBI Entrez gene database. Probes passing the tests were analysed by *Ingenuity Pathway Analysis* (*IPA*) software which performs a gene set enrichment analysis and groups genes by biological functional categories and canonical pathways. The *p*-*value* reflects the significance of the enrichment of input genes in each functional category or pathway.

### Western blot

Cells were plated in T75 tissue culture flasks in complete medium and grown to semi-confluence, then were treated for 24 hours with medium containing or not 10 μM D6. Cells were harvested using cell-scraper and cold PBS w/o Ca^++^/Mg^++^ (Sigma-Aldrich, St. Louis, MO, USA), and then lysed with lysis buffer (50 mM Tris HCl pH 7.5, 5 mM EDTA, 100 mM NaCl, 1 mM Na_4_P_2_O_7_, 1% Triton X100) plus protease inhibitor cocktail (Sigma-Aldrich, St. Louis, MO, USA). Protein concentration was determined by the QuantiPro BCA Assay Kit (Sigma-Aldrich, St. Louis, MO, USA). Protein lysates (30 μg proteins per lane) were resolved onto 10% or 12% NuPAGE® Novex® Bis-Tris Mini Gels and transferred by iBlot™ Dry Blotting System to iBlot® Gel Transfer Stacks Nitrocellulose, Mini (all from Invitrogen). The membranes were then incubated with primary antibodies over night at 4°C. Primary antibodies used were: mouse monoclonal antibodies against p21, cyclin B1, cdc25, PI3-kinase p85β, c-kit, GAPDH or rabbit polyclonal antibodies against GADD45A, Noxa, p-AKT 1/2/3 (Ser 474), all from Santa Cruz Biotechnology Inc. Detection was achieved by HRP-conjugated anti-mouse (Chemicon, Billerica, MA, USA, 1:10,000) or HRP-conjugated anti-rabbit (Santa Cruz Biotech, 1:1,000,000) antibodies. Immune complexes were visualized with the use of an enhanced chemiluminescence system (ECL Advance™, Amersham International). Protein levels were quantified by ImageJ software [58] and normalized over (ratio) the GAPDH protein levels used as internal control.

## Competing interests

The authors declare that they have not competing interests.

## Authors’ contributions

CR conceived and designed the study, coordinated it, participated to cell cultures, RNA extractions, western blot analysis, and drafted the manuscript. MF and CF together carried out statistical analysis of gene expression profile analysis data and contributed to final drafting of manuscript. AC carried out cell cultures, RNA extraction, gene expression profile analysis and contributed to statistical analysis. TC carried out gene expression profile analysis. IS and SC performed cell cultures, D6 cellular uptake assays and western blot analysis. VN participated to cell cultures and cell cycle progression analysis. GG carried out cytofluorimetric assays. AE carried out LC/MS analysis. MAD and DF performed the chemical synthesis of the curcumin-related biphenyl compound D6. GP contributed to the final drafting and critical revision of the manuscript. MP carried out cell cultures, western blot analysis and participated in study design, coordination and final drafting of the manuscript. All authors read and approved the final manuscript.

## Supplementary Material

Additional file 1 **Melanoma cells: gene expression profile results.** Excel file composed of three sheet: 1-A, 1-B, 1-C. 1-A: List of 1173 probes differentially expressed in LB24Dagi melanoma cells after 16 hours of exposure to 10 μM D6. The list resulted from the statistical analysis performed by using the BRB-Array Tools as described in the Methods section. Data were filtered by fold change (FC) considering as differentially expressed probes only those showing 0.5 > FC > 2. In column C (Gene symbol) hyperlink to gene annotations from the NCBI Entrez gene database are supplied. 1-B: List of bio-functional categories, identified by Ingenuity Pathway Analysis (IPA) software by analysing the 1173 transcripts modulated in 10 μM D6 treated LB24Dagi melanoma cells (column A). Function annotations (column B), number of genes of the input list involved in each functional category (column E) and their symbol (column F) are shown in the table. Right-tailed Fisher’s exact test has been performed to calculate a *p*-*value* (column C) determining the probability that each biological function assigned to the selected transcripts was due to chance. *p*-*values* were adjusted for multiple comparisons using the Benjamini-Hochberg correction (column D). 1-C: List of canonical pathways, identified by IPA software by analysing the 1173 transcripts modulated in 10 μM D6 treated LB24Dagi melanoma cells (column A). *p*-*values* (calculated using Fisher’s exact test) reflect the probability that the association between the genes in the dataset and the canonical pathway is explained by chance alone (column B). The ratios in column C represent the number of molecules in a given pathway that meet cut-off criteria, divided by total number of molecules that make up that pathway. Genes of the input list involved in each pathway are shown in column D. Click here for file

Additional file 2 **Fibroblasts: gene expression profile results.** Excel file composed of three sheets: 2-A, 2-B, 2-C. 2-A: List of 1883 probes differentially expressed in BJ normal fibroblasts after 16 hours of exposure to 10 μM D6. The list resulted from the statistical analysis performed by using the BRB-Array Tools as described in the Methods section. Data were filtered by fold change (FC) considering as differentially expressed probes only those showing 0.5 > FC > 2. In column C (Gene symbol) hyperlink to gene annotations from the NCBI Entrez gene database are supplied. 2-B: List of bio-functional categories, identified by Ingenuity Pathway Analysis (IPA) software by analysing the 1883 transcripts modulated in 10 μM D6 treated BJ normal fibroblasts (column A). Function annotations (column B), number of genes of the input list involved in each functional category (column E) and their symbol (column F) are shown in the table. Right-tailed Fisher’s exact test has been performed to calculate a *p*-*value* (column C) determining the probability that each biological function assigned the selected transcripts was due to chance. *p*-*values* were adjusted for multiple comparisons using the Benjamini-Hochberg correction (column D). 2-C: List of canonical pathways, identified by IPA software by analysing the 1883 transcripts modulated in 10 μM D6 treated BJ normal fibroblasts (column A). *p*-*values* (calculated using Fisher’s exact test) reflect the probability that the association between the genes in the dataset and the canonical pathway is explained by chance alone (column B). The ratios in column C represent the number of molecules in a given pathway that meet cut-off criteria, divided by total number of molecules that make up that pathway. Genes of the input list involved in each pathway are shown in column D. Click here for file

Additional file 3 **Cell cycle: G2/M DNA checkpoint regulation.** pdf file elaborated by Ingenuity Pathway Analysis (IPA) software. The diagram schematizes the “Cell cycle: G2/M DNA checkpoint regulation” pathway (n.° 4 in Table 3) found to be significantly down-regulated in D6 treated melanoma cells. Up-regulated genes are represented in red gradations, down-regulated genes in green gradations. Colour intensity for each gene is proportional to its FC value. Click here for file

Additional file 4 **p53 signalling pathway. pdf file, elaborated by Ingenuity Pathway Analysis (IPA) software.** The diagram schematizes the “p53 signalling” pathway (n.° 5 in Table 3) found to be significantly induced in D6 treated melanoma cells. Up-regulated genes are represented in red gradations, down-regulated genes in green gradations. Colour intensity for each gene is proportional to its FC value. Click here for file

Additional file 5 **Hereditary breast cancer signalling.** pdf file elaborated by Ingenuity Pathway Analysis (IPA) software. The diagram schematizes the “Hereditary breast cancer signalling” pathway (n.° 10 in Table 3) found to be significantly down-regulated in D6 treated melanoma cells. Up-regulated genes are represented in red gradations, down-regulated genes in green gradations. Colour intensity for each gene is proportional to its FC value. Click here for file

Additional file 6 **ATM signalling. pdf file elaborated by Ingenuity Pathway Analysis (IPA) software.** The diagram schematizes the “ATM signalling” pathway (n.° 11 in Table 3) found to be significantly induced in D6 treated melanoma cells. Up-regulated genes are represented in red gradations, down-regulated genes in green gradations. Colour intensity for each gene is proportional to its FC value. Click here for file

Additional file 7 **Role of BRCA1 in DNA damage response.** pdf file elaborated by Ingenuity Pathway Analysis (IPA) software. The diagram schematizes the “Role of BRCA1 in DNA damage response” pathway (n.° 26 in Table 3) found to be significantly induced in D6 treated melanoma cells. Up-regulated genes are represented in red gradations, down-regulated genes in green gradations. Colour intensity for each gene is proportional to its FC value. Click here for file

Additional file 8 **Role of CHK proteins in cell cycle checkpoint control.** pdf file elaborated by Ingenuity Pathway Analysis (IPA) software. The diagram schematizes the “Role of CHK proteins in cell cycle checkpoint control” pathway (n.° 27 in Table 3) found to be significantly down-regulated in D6 treated melanoma cells. Up-regulated genes are represented in red gradations, down-regulated genes in green gradations. Colour intensity for each gene is proportional to its FC value. Click here for file

Additional file 9 **Mitotic roles of Polo-like kinase.** pdf file elaborated by Ingenuity Pathway Analysis (IPA) software. The diagram schematizes the “Mitotic roles of Polo-like kinase” pathway (n.° 8 in Table 3) found to be significantly down-regulated in D6 treated melanoma cells. Up-regulated genes are represented in red gradations, down-regulated genes in green gradations. Colour intensity for each gene is proportional to its FC value. Click here for file

Additional file 10 **Cyclins and cell cycle regulation. pdf file elaborated by Ingenuity Pathway Analysis (IPA) software.** The diagram schematizes the “Cyclins and cell cycle regulation” pathway (n.° 13 in Table 3) found to be significantly down-regulated in D6 treated melanoma cells. Up-regulated genes are represented in red gradations, down-regulated genes in green gradations. Colour intensity for each gene is proportional to its FC value. Click here for file

Additional file 11 **Molecular mechanisms of cancer. pdf file elaborated by Ingenuity Pathway Analysis (IPA) software.** The diagram schematizes the “Molecular mechanisms of cancer” pathway (n.° 23 in Table 3) found to be significant in D6 treated melanoma cells. Up-regulated genes are represented in red gradations, down-regulated genes in green gradations. Colour intensity for each gene is proportional to its FC value. Click here for file

Additional file 12 **Cell cycle: G1/S Checkpoint regulation.** pdf file elaborated by Ingenuity Pathway Analysis (IPA) software. The diagram schematizes the “Cell cycle: G1/S Checkpoint regulation” pathway (n.° 25 in Table 3) found to be significantly down-regulated in D6 treated melanoma cells. Up-regulated genes are represented in red gradations, down-regulated genes in green gradations. Colour intensity for each gene is proportional to its FC value. Click here for file
