# Supplementary figures and images for: Molecular changes induced by the curcumin analogue D6 in human melanoma cells
Source: Mol Cancer. 2013 May 4;12:37. doi: 10.1186/1476-4598-12-37 (PMC3651720; doi:10.1186/1476-4598-12-37)

# Cell Cycle: G2/M DNA Damage Checkpoint Regulation

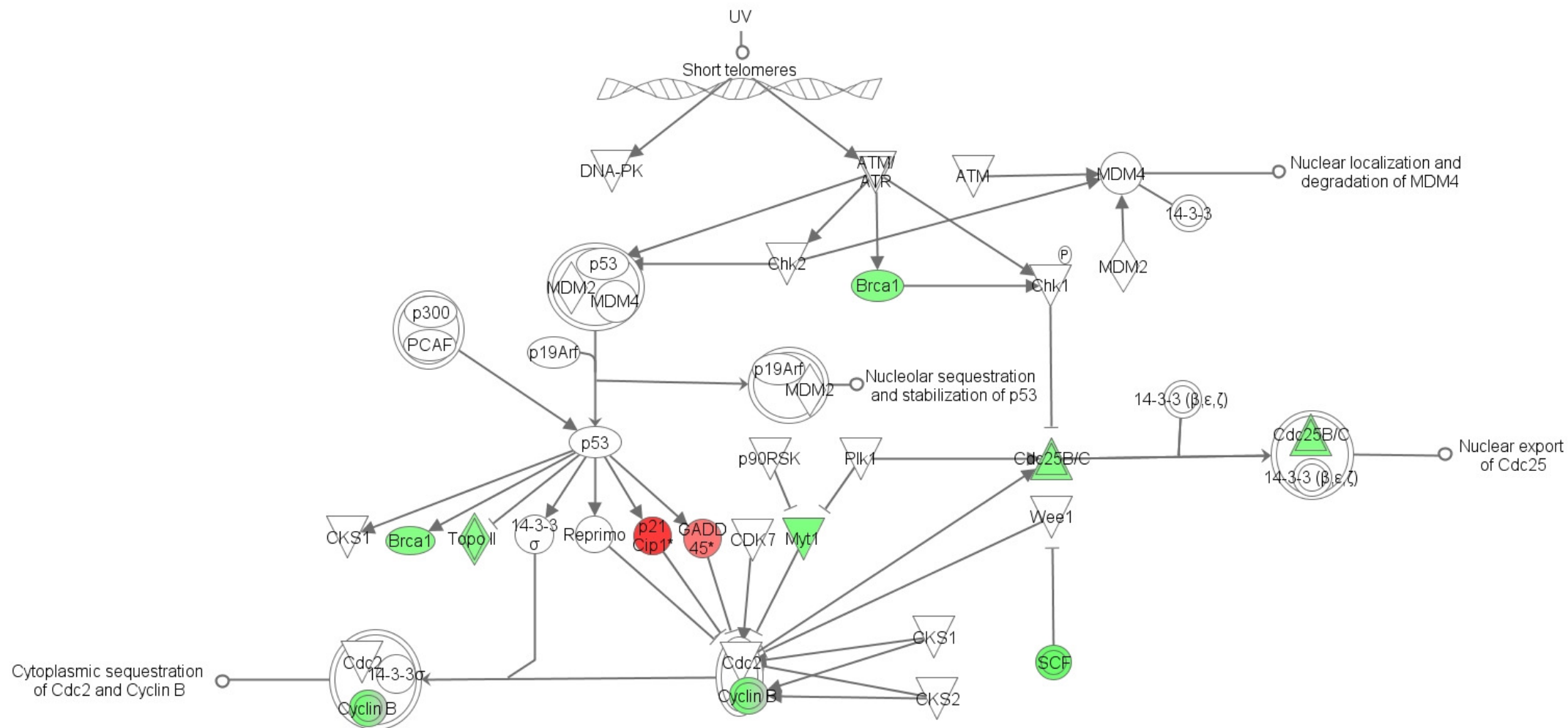

Supplement: Additional file 3 — Cell cycle: G2/M DNA checkpoint regulation. pdf file elaborated by Ingenuity Pathway Analysis (IPA) software. The diagram schematizes the “Cell cycle: G2/M DNA checkpoint regulation” pathway (n.° 4 in Table 3) found to be significantly down-regulated in D6 treated melanoma cells. Up-regulated genes are represented in red gradations, down-regulated genes in green gradations. Colour intensity for each gene is proportional to its FC value. [file 1476-4598-12-37-S3.pdf]

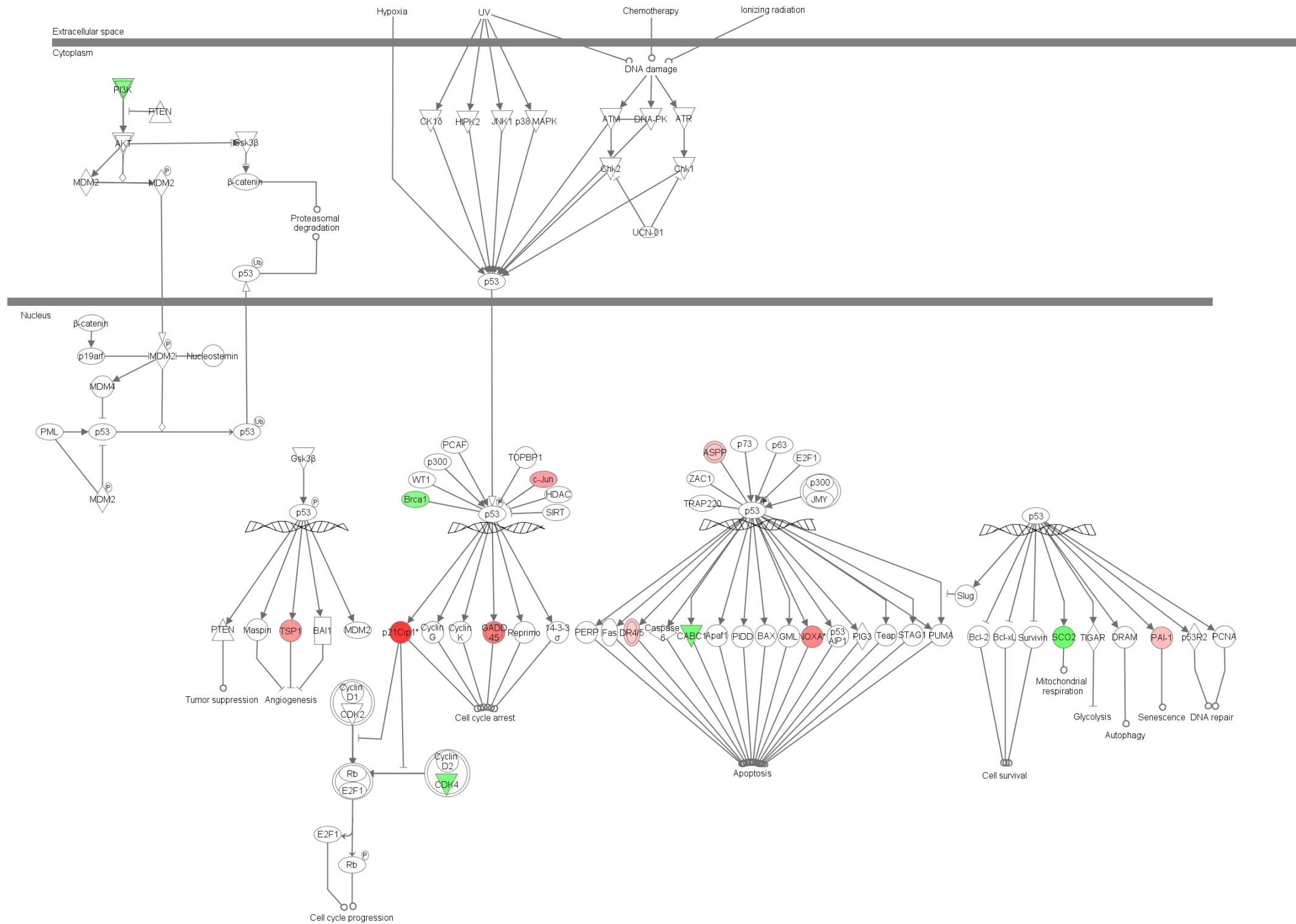

Supplement: Additional file 4 — p53 signalling pathway. pdf file, elaborated by Ingenuity Pathway Analysis (IPA) software. The diagram schematizes the “p53 signalling” pathway (n.° 5 in Table 3) found to be significantly induced in D6 treated melanoma cells. Up-regulated genes are represented in red gradations, down-regulated genes in green gradations. Colour intensity for each gene is proportional to its FC value. [file 1476-4598-12-37-S4.pdf]

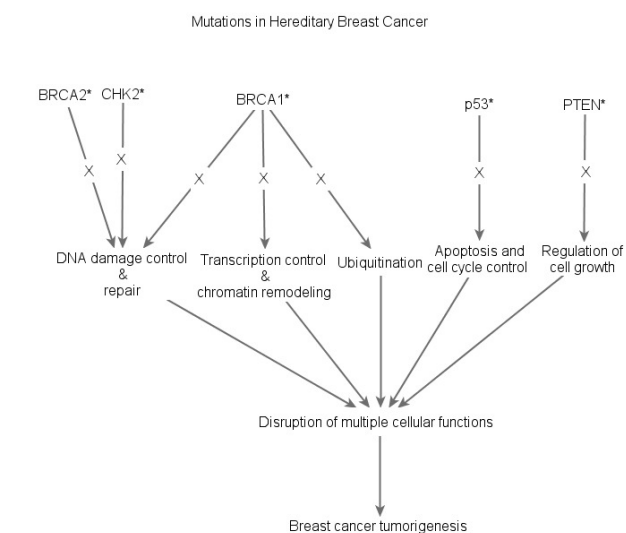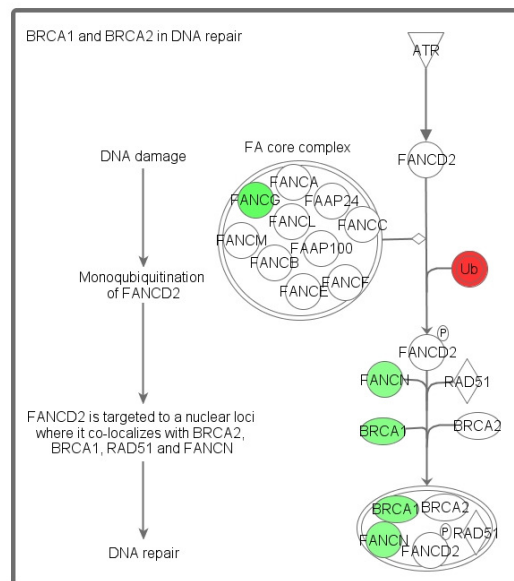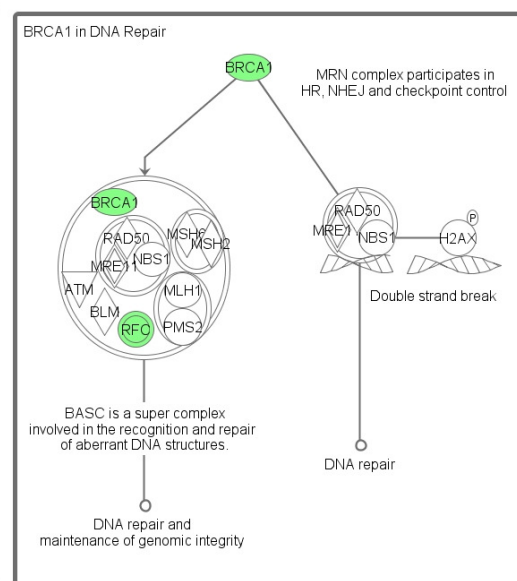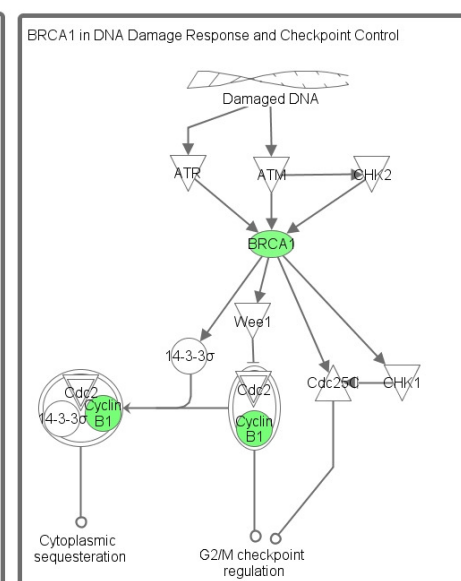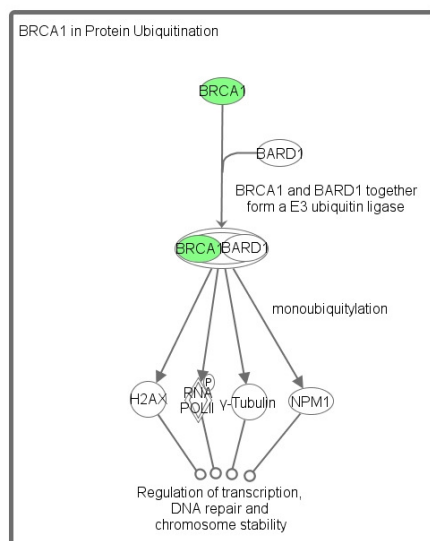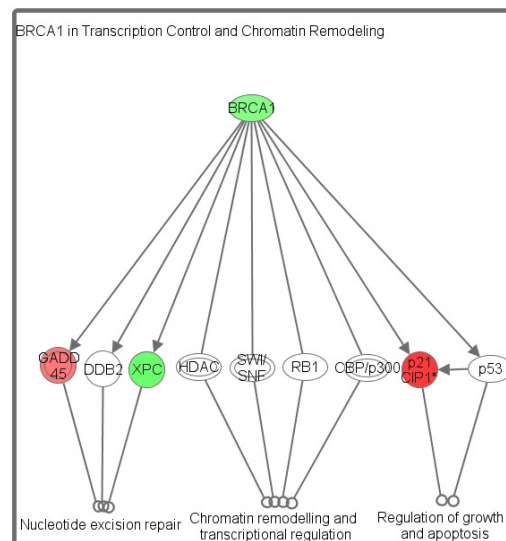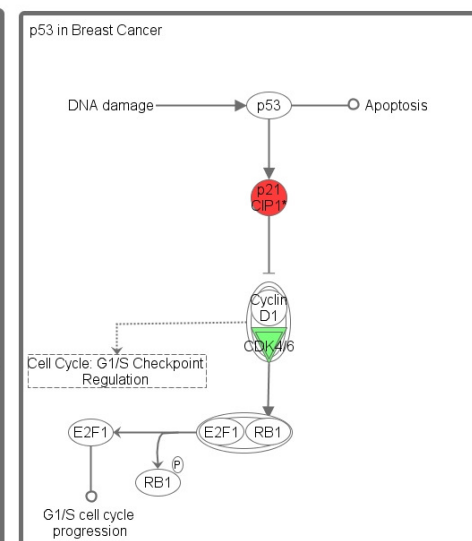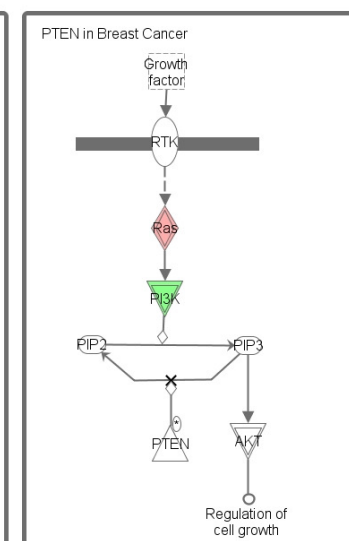

Supplement: Additional file 5 — Hereditary breast cancer signalling. pdf file elaborated by Ingenuity Pathway Analysis (IPA) software. The diagram schematizes the “Hereditary breast cancer signalling” pathway (n.° 10 in Table 3) found to be significantly down-regulated in D6 treated melanoma cells. Up-regulated genes are represented in red gradations, down-regulated genes in green gradations. Colour intensity for each gene is proportional to its FC value. [file 1476-4598-12-37-S5.pdf]

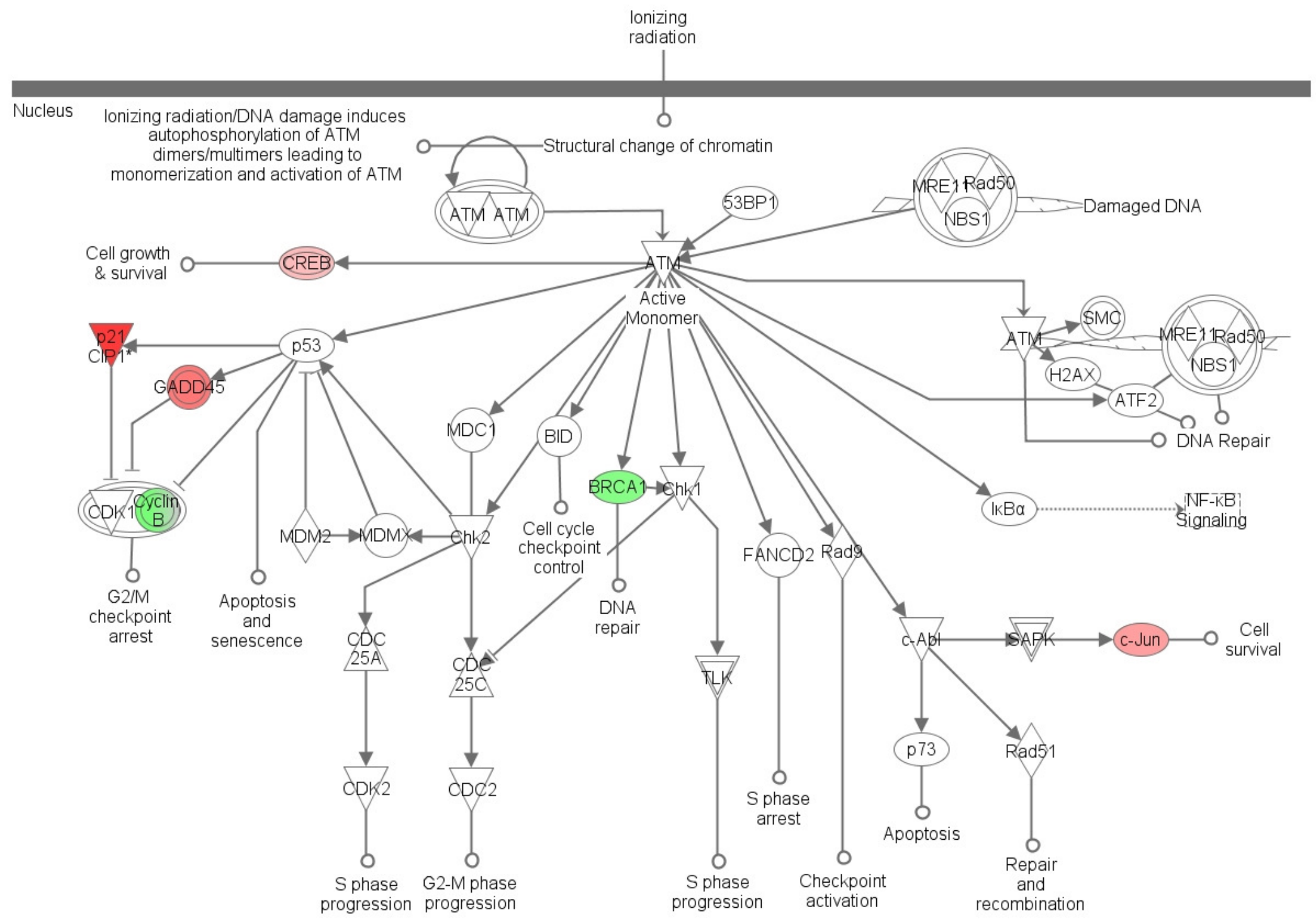

Supplement: Additional file 6 — ATM signalling. pdf file elaborated by Ingenuity Pathway Analysis (IPA) software. The diagram schematizes the “ATM signalling” pathway (n.° 11 in Table 3) found to be significantly induced in D6 treated melanoma cells. Up-regulated genes are represented in red gradations, down-regulated genes in green gradations. Colour intensity for each gene is proportional to its FC value. [file 1476-4598-12-37-S6.pdf]

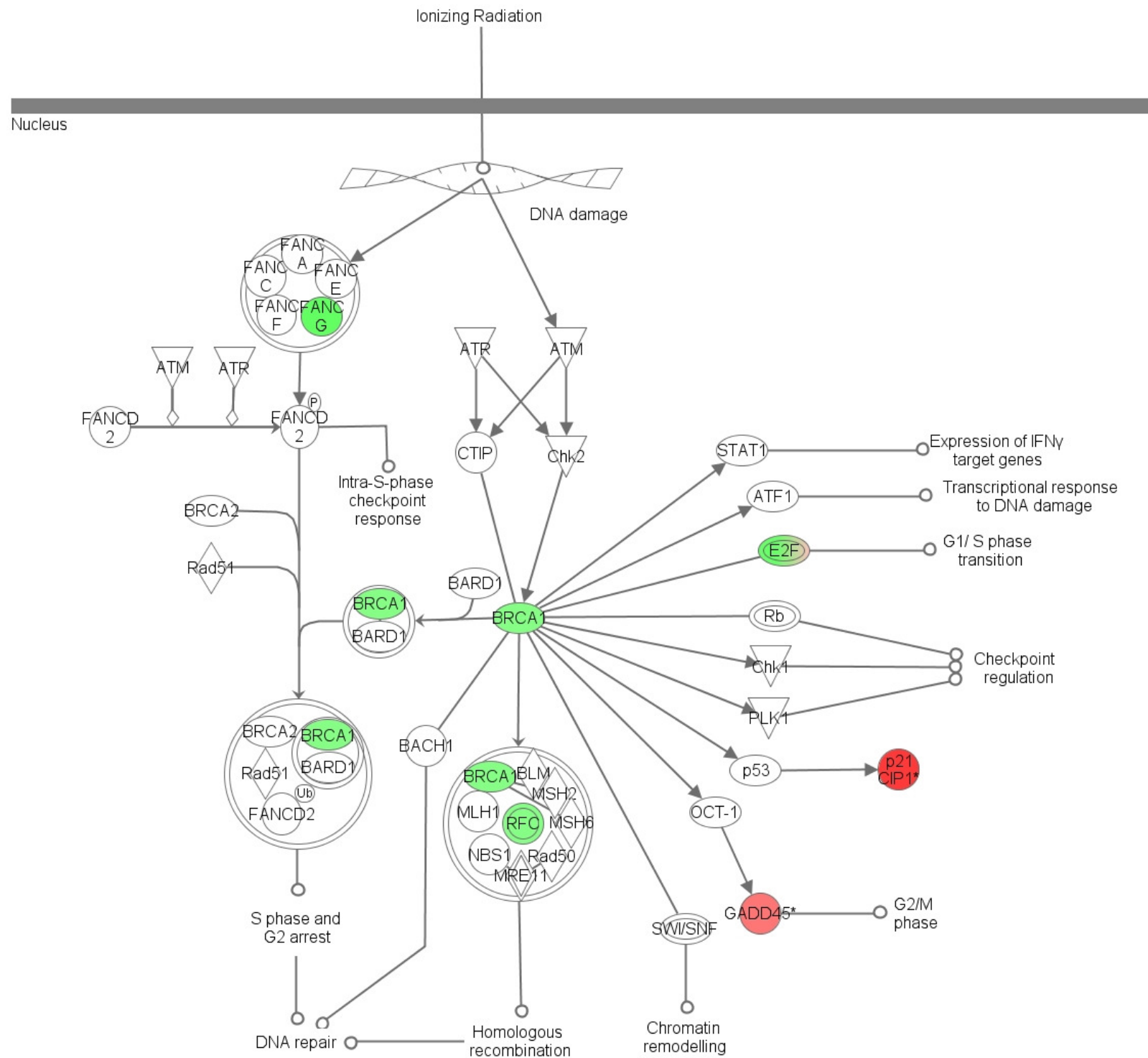

Supplement: Additional file 7 — Role of BRCA1 in DNA damage response. pdf file elaborated by Ingenuity Pathway Analysis (IPA) software. The diagram schematizes the “Role of BRCA1 in DNA damage response” pathway (n.° 26 in Table 3) found to be significantly induced in D6 treated melanoma cells. Up-regulated genes are represented in red gradations, down-regulated genes in green gradations. Colour intensity for each gene is proportional to its FC value. [file 1476-4598-12-37-S7.pdf]

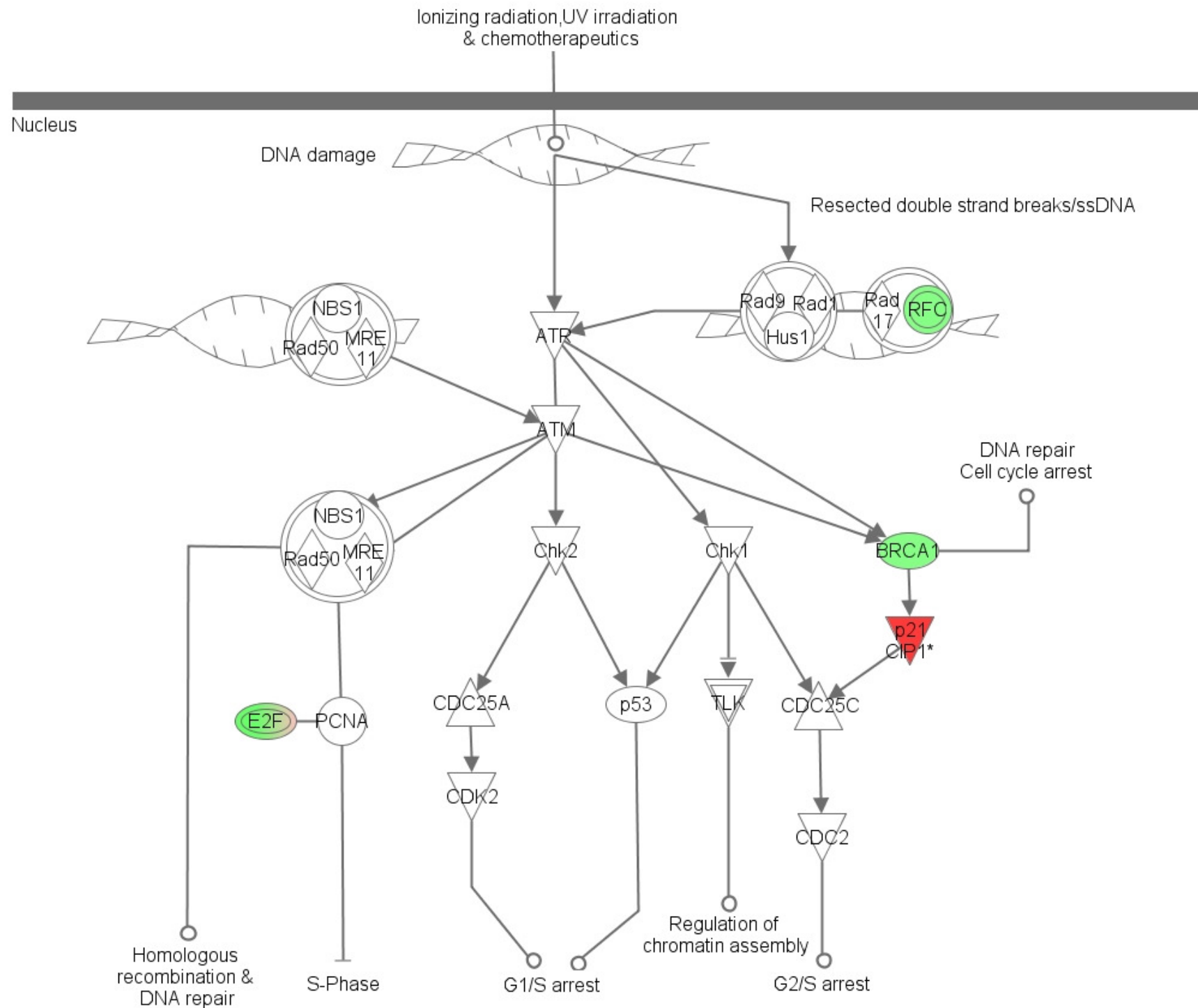

Supplement: Additional file 8 — Role of CHK proteins in cell cycle checkpoint control. pdf file elaborated by Ingenuity Pathway Analysis (IPA) software. The diagram schematizes the “Role of CHK proteins in cell cycle checkpoint control” pathway (n.° 27 in Table 3) found to be significantly down-regulated in D6 treated melanoma cells. Up-regulated genes are represented in red gradations, down-regulated genes in green gradations. Colour intensity for each gene is proportional to its FC value. [file 1476-4598-12-37-S8.pdf]

# Mitotic Roles of Polo-Like Kinase

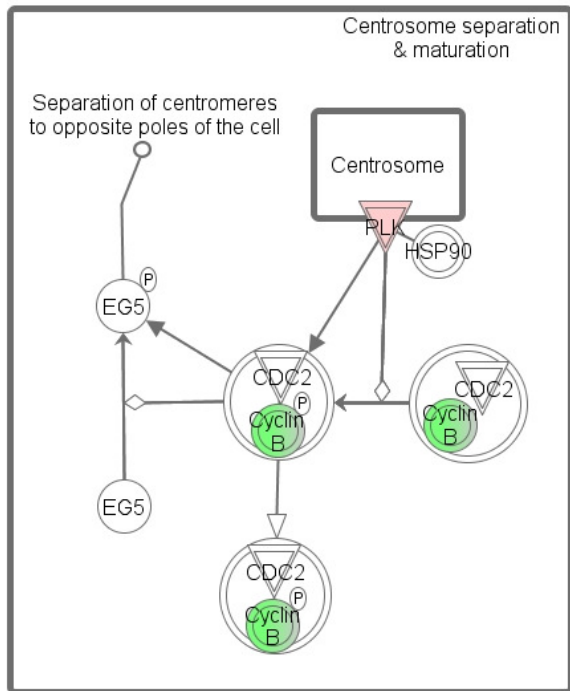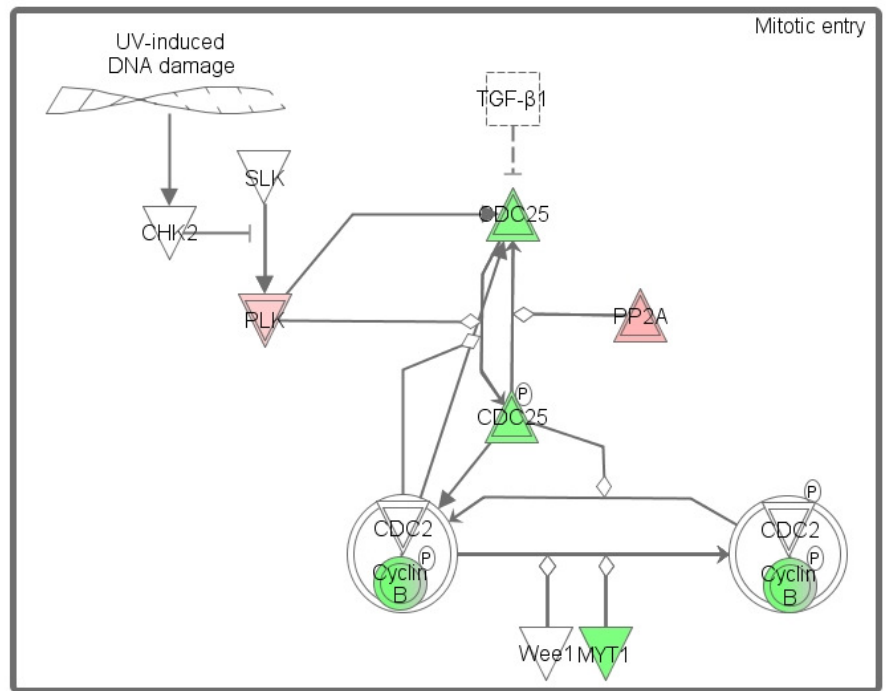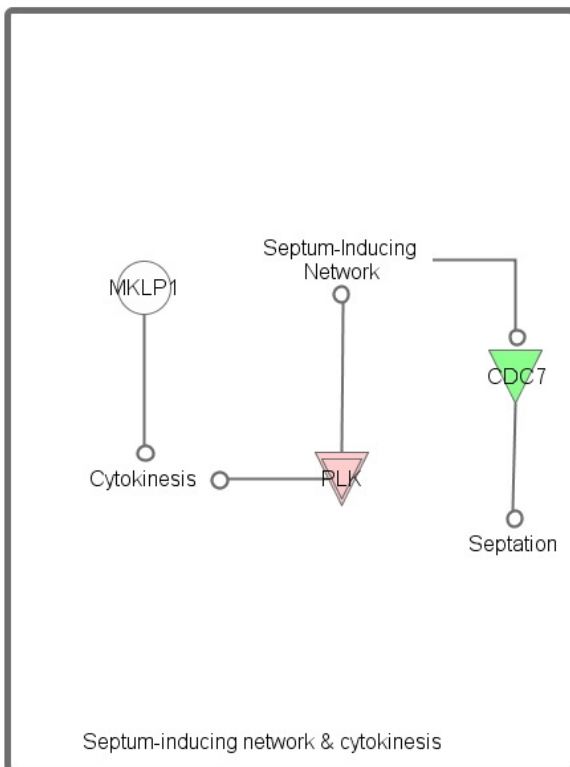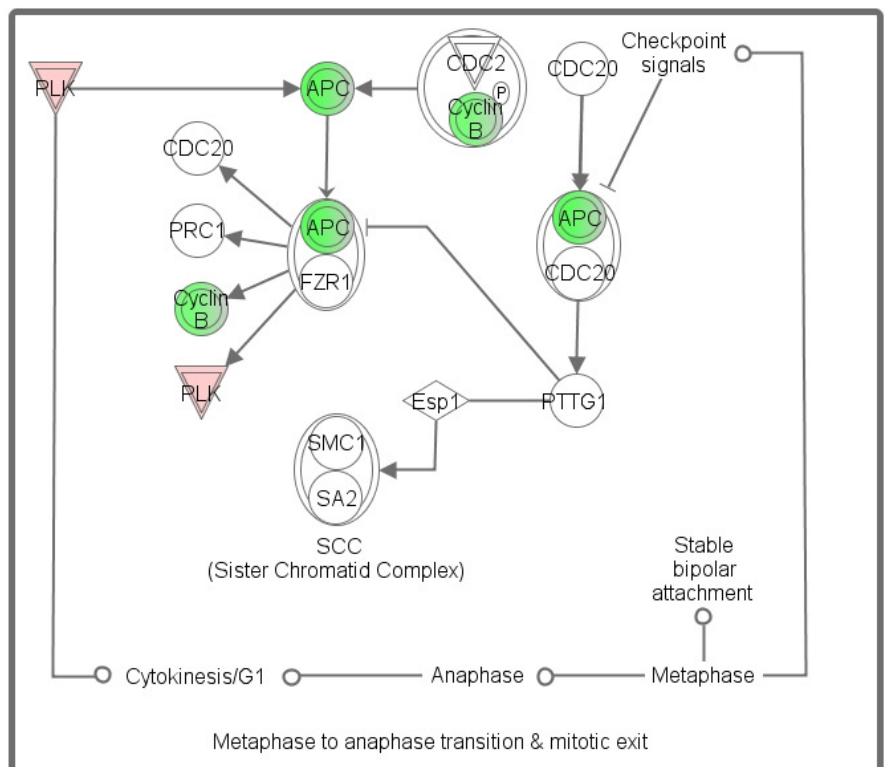

Supplement: Additional file 9 — Mitotic roles of Polo-like kinase. pdf file elaborated by Ingenuity Pathway Analysis (IPA) software. The diagram schematizes the “Mitotic roles of Polo-like kinase” pathway (n.° 8 in Table 3) found to be significantly down-regulated in D6 treated melanoma cells. Up-regulated genes are represented in red gradations, down-regulated genes in green gradations. Colour intensity for each gene is proportional to its FC value. [file 1476-4598-12-37-S9.pdf]

© 2000-2012 Ingenuity Systems, Inc. All rights reserved.

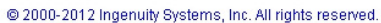

Supplement: Additional file 10 — Cyclins and cell cycle regulation. pdf file elaborated by Ingenuity Pathway Analysis (IPA) software. The diagram schematizes the “Cyclins and cell cycle regulation” pathway (n.° 13 in Table 3) found to be significantly down-regulated in D6 treated melanoma cells. Up-regulated genes are represented in red gradations, down-regulated genes in green gradations. Colour intensity for each gene is proportional to its FC value. [file 1476-4598-12-37-S10.pdf]

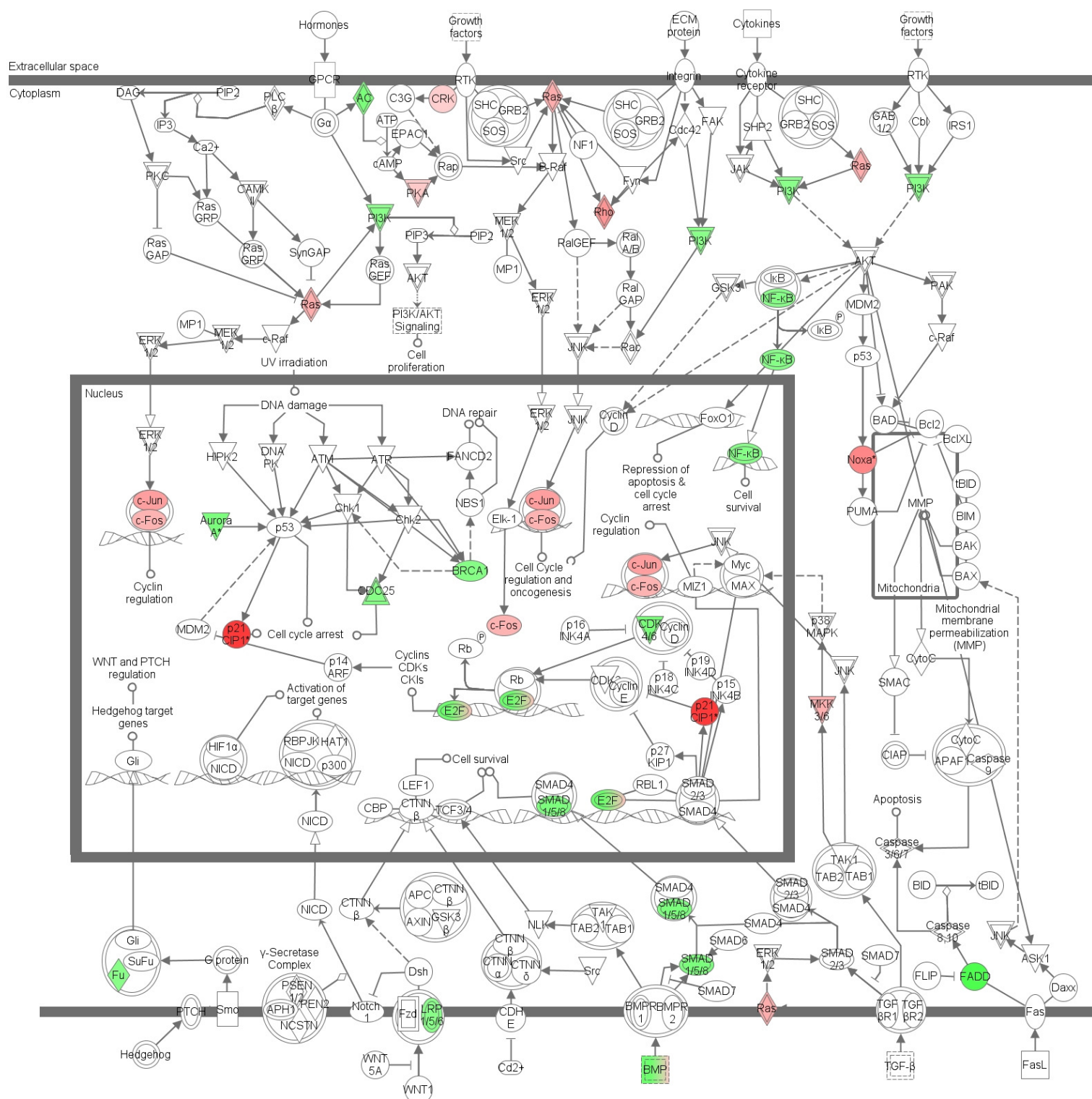

Supplement: Additional file 11 — Molecular mechanisms of cancer. pdf file elaborated by Ingenuity Pathway Analysis (IPA) software. The diagram schematizes the “Molecular mechanisms of cancer” pathway (n.° 23 in Table 3) found to be significant in D6 treated melanoma cells. Up-regulated genes are represented in red gradations, down-regulated genes in green gradations. Colour intensity for each gene is proportional to its FC value. [file 1476-4598-12-37-S11.pdf]

# Cell Cycle: G1/S Checkpoint Regulation

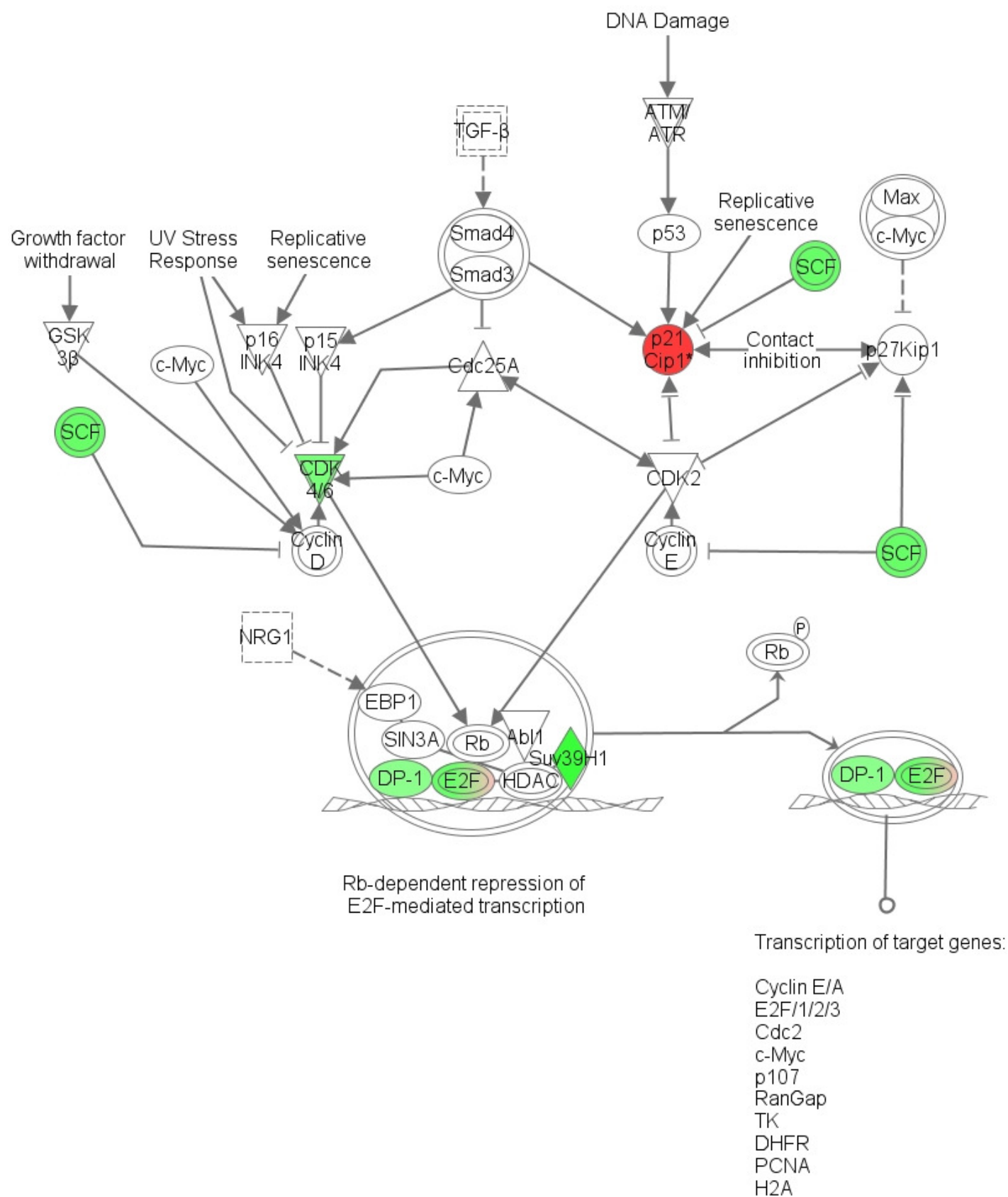

Supplement: Additional file 12 — Cell cycle: G1/S Checkpoint regulation. pdf file elaborated by Ingenuity Pathway Analysis (IPA) software. The diagram schematizes the “Cell cycle: G1/S Checkpoint regulation” pathway (n.° 25 in Table 3) found to be significantly down-regulated in D6 treated melanoma cells. Up-regulated genes are represented in red gradations, down-regulated genes in green gradations. Colour intensity for each gene is proportional to its FC value. [file 1476-4598-12-37-S12.pdf]
